# A vital role for PICK1 in the differential regulation of metabotropic glutamate receptor internalization and synaptic AMPA receptor endocytosis

**DOI:** 10.1016/j.jbc.2023.104837

**Published:** 2023-05-18

**Authors:** Namrata Ramsakha, Prachi Ojha, Subhajit Pal, Sanjeev Routh, Ami Citri, Samarjit Bhattacharyya

**Affiliations:** 1 Department of Biological Sciences, Indian Institute of Science Education and Research Mohali, SAS Nagar, Punjab, India; 2 The Edmond and Lily Safra Center for Brain Sciences, The Hebrew University of Jerusalem, Edmond J. Safra Campus, Jerusalem, Israel; 3 Institute of Life Sciences, The Hebrew University of Jerusalem, Edmond J. Safra Campus, Jerusalem, Israel; 4 Program in Child and Brain Development, Canadian Institute for Advanced Research, MaRS Centre, Toronto, Ontario, Canada

**Keywords:** endocytosis, G protein-coupled receptor (GPCR), metabotropic glutamate receptor (mGluR), protein interacting with C kinase 1 (PICK1), receptor internalization, synaptic plasticity, trafficking

## Abstract

Group I metabotropic glutamate receptors (mGluRs) play important roles in many neuronal processes and are believed to be involved in synaptic plasticity underlying the encoding of experience, including classic paradigms of learning and memory. These receptors have also been implicated in various neurodevelopmental disorders, such as Fragile X syndrome and autism. Internalization and recycling of these receptors in the neuron are important mechanisms to regulate the activity of the receptor and control the precise spatiotemporal localization of these receptors. Applying a “molecular replacement” approach in hippocampal neurons derived from mice, we demonstrate a critical role for protein interacting with C kinase 1 (PICK1) in regulating the agonist-induced internalization of mGluR1. We show that PICK1 specifically regulates the internalization of mGluR1, but it does not play any role in the internalization of the other member of group I mGluR family, mGluR5. Various regions of PICK1 *viz*., the N-terminal acidic motif, PDZ domain, and BAR domain play important roles in the agonist-mediated internalization of mGluR1. Finally, we demonstrate that PICK1-mediated internalization of mGluR1 is critical for the resensitization of the receptor. Upon knockdown of endogenous PICK1, mGluR1s stayed on the cell membrane as inactive receptors, incapable of triggering the MAP kinase signaling. They also could not induce AMPAR endocytosis, a cellular correlate for mGluR-dependent synaptic plasticity. Thus, this study unravels a novel role for PICK1 in the agonist-mediated internalization of mGluR1 and mGluR1-mediated AMPAR endocytosis that might contribute to the function of mGluR1 in neuropsychiatric disorders.

Glutamate is a major excitatory neurotransmitter in the central nervous system, which transduces its signal through ionotropic and metabotropic glutamate receptors (mGluRs) (1, 2). Group I mGluRs comprising of mGluR1 and mGluR5 are localized at the perisynaptic region of the postsynaptic neuron. They are thought to play an important role in multiple forms of experience-dependent synaptic plasticity, including learning and memory (3, 4). Activity-dependent changes in the strength of excitatory synapses result from the mGluR-regulated trafficking of AMPARs to and away from the synapses (3, 4, 5, 6). These receptors have also been implicated in various neuropsychiatric disorders such as schizophrenia, fragile X syndrome, and autism (3, 7, 8, 9). Similar to many other GPCRs, trafficking plays a crucial role in controlling the spatiotemporal localization and activity of these receptors. Following agonist binding, group I mGluRs undergo desensitization followed by rapid internalization (2, 4, 10, 11, 12). Subsequently, they recycle back to the cell surface, which is the mechanism for “resensitization” of these receptors (13, 14, 15). Despite the obvious significance of this trafficking for receptor function, the protein machineries that control group I mGluR internalization and mGluR-mediated AMPAR endocytosis are not fully characterized.

Protein interacting with C kinase 1 (PICK1) is a small protein (416 aa) localized in the postsynaptic density (PSD) and containing an N-terminal PSD-95/DLG/ZO1 (PDZ) domain and a C-terminal Bin/amphiphysin/Rvs (BAR) domain (16). PICK1 interacts with most of its partner proteins *via* its PDZ domain (17, 18, 19, 20, 21). The BAR domain of PICK1 dimerizes and forms a crescent shaped structure that binds and facilitates emergence of membrane curvatures which helps in the formation of vesicles (22, 23). Through its BAR domain, PICK1 also interacts with members of the SNARE-dependent membrane fusion machinery, actin, GRIP1, and charged membrane lipids (20, 24, 25, 26). PICK1 contains two acidic stretches, one at the N terminus and another at the C terminus of the protein, which bind calcium (27, 28). Furthermore, PICK1 plays crucial roles in AMPAR trafficking and synaptic plasticity (22, 26, 27, 28, 29). Thus, considering the binding partners with which PICK1 interacts and its role in synaptic plasticity, we hypothesized that PICK1 might play an important role in group I mGluR internalization, as well as in mGluR-mediated AMPAR endocytosis.

We performed our study using a molecular replacement strategy that allowed shRNA-mediated acute knockdown of endogenous PICK1 and replacement of the endogenous form with various mutant forms in primary hippocampal neurons. We show here that PICK1 plays a contrasting role in the agonist-mediated internalization of mGluR1 and mGluR5. Acute knockdown of endogenous PICK1 decreased the surface expression and inhibited the agonist-induced endocytosis of mGluR1. PICK1 was observed to interact with mGluR1. On the other hand, although knockdown of PICK1 decreased the surface expression of mGluR5, it did not inhibit the agonist-mediated endocytosis of mGluR5. The PDZ domain and BAR domain of PICK1 were critical for the internalization of mGluR1. We also found that orientation of the amino acid sequence at the N-terminal acidic region of PICK1 was important for the agonist-mediated endocytosis of mGluR1. Finally, we show that PICK1 specifically regulates mGluR1-mediated signaling and mGluR1-mediated AMPAR internalization, the cellular correlate of mGluR1-dependent synaptic plasticity. Thus, our study unravels a previously uncharacterized role for PICK1 in the internalization of mGluR1 and mGluR1-mediated AMPAR endocytosis, with anticipated clinical relevance to the function of mGluR1 in neuropsychiatric disorders.

## Results

### PICK1 regulates the agonist-mediated internalization of mGluR1

**Figure 1:**
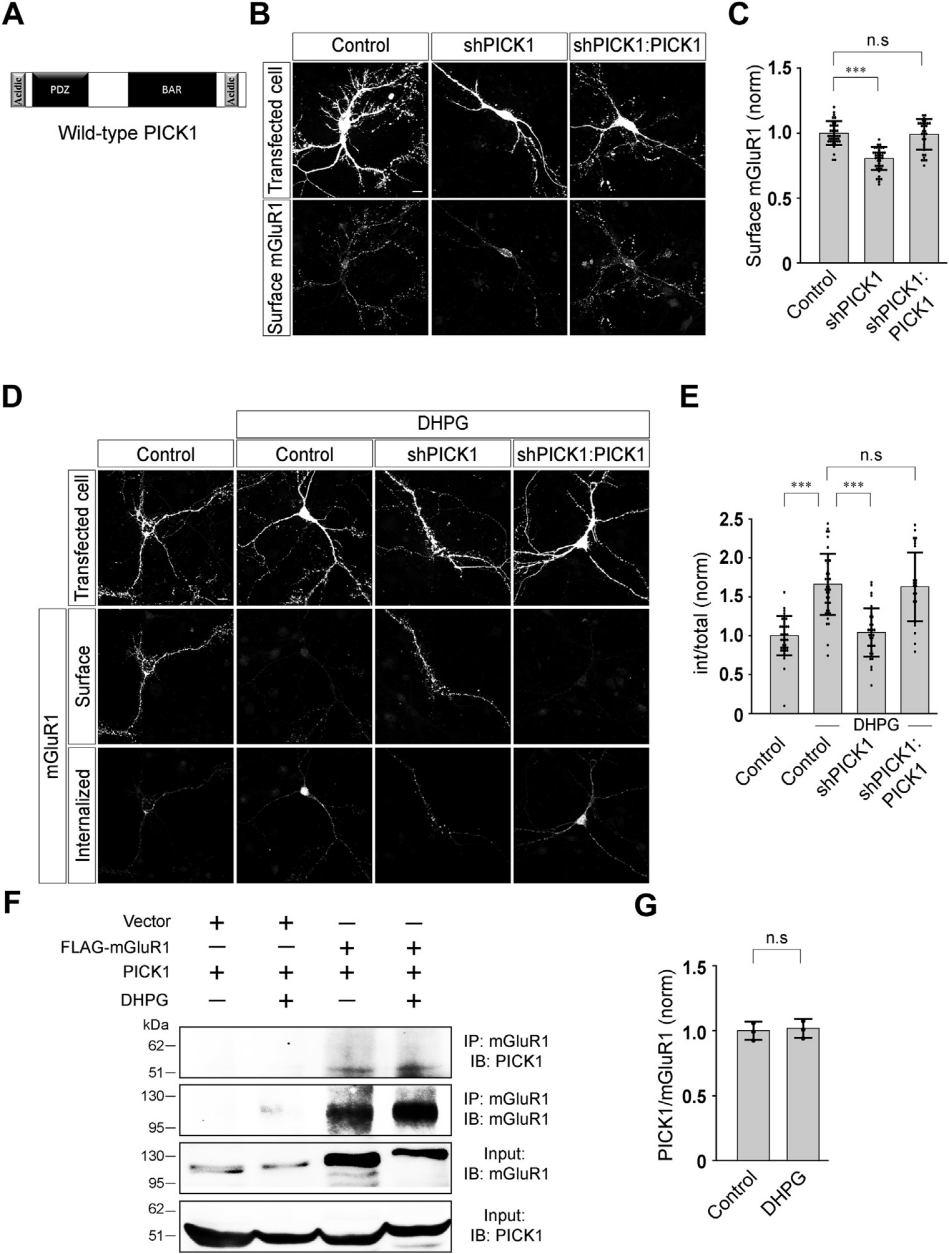
**Knockdown of endogenous PICK1 decreases the surface expression and inhibits the agonist-mediated internalization of mGluR1.***A*, schematic depicting the domain structure of full-length PICK1. *B* and *C*, representative images (*B*) and quantitation (*C*) of surface myc-mGluR1 showing that due to the knockdown of the endogenous PICK1 by shPICK1, surface myc-mGluR1 expression was reduced compared to control cells and this reduction was rescued by the expression of WT PICK1 (N: control = 39; shPICK1 = 40; shPICK1:PICK1 = 32). *D* and *E*, representative images (*D*) and quantitation (*E*) of 100 μM R,S-DHPG–induced internalization of myc-mGluR1 suggesting that knockdown of endogenous PICK1 led to the inhibition of agonist-mediated endocytosis of myc-mGluR1, and expression of the WT PICK1 replacement construct rescued the normal endocytosis of the receptor (N: control = 40; control + DHPG = 41; shPICK1 + DHPG = 34; shPICK1:PICK1 + DHPG = 30). *F* and *G*, co-immunoprecipitation assay (*F*) and quantitation of the co-immunoprecipitation assays (*G*) demonstrating that PICK1 interacts with FLAG-mGluR1, and the interaction did not increase upon application of 100 μM R,S-DHPG (N = 3). Results are presented as means ± SD. Scale bar represents 10 μm. ∗∗∗*p* < 0.001; n.s, *p* > 0.05. DHPG, 3, 5-dihydroxyphenylglycine; mGluR, metabotropic glutamate receptor; PICK1, protein interacting with C kinase 1.

Similar to many other GPCRs, group I mGluRs also undergo rapid internalization following agonist exposure (13, 30, 31, 32, 33). The cellular and molecular mechanisms that govern the agonist-mediated endocytosis of group I mGluRs are poorly understood. Like many other GPCRs, group I mGluRs are also tightly regulated by a macromolecular protein complex at the postsynaptic membrane, known as PSD. One member of the PSD is PICK1. It acts like an adapter molecule as it binds to a variety of membrane proteins and organizes the subcellular localization and surface expression of many proteins (16, 18, 20, 25, 26). In order to investigate whether PICK1 could play any role in the agonist-mediated internalization of group I mGluRs, we studied the agonist-induced internalization of myc-mGluR1 by using a “molecular replacement” strategy that allows simultaneous shRNA-mediated acute knockdown of endogenous PICK1 and expression of various mutant forms of recombinant PICK1 in primary hippocampal neurons. This approach offers two important advantages. First, the possibility of compensatory adaptations during synaptogenesis and synapse maturation due to the absence of the protein of interest are minimal. Second, the function of the protein of interest can be studied without the necessity to obtain a dominant effect as required by a standard overexpression approach. We used an shRNA against PICK1 that has been reported to efficiently knock down endogenous PICK1 in primary hippocampal neurons and has been employed successfully in an earlier study (28). The rescue of the effect of shPICK1 knockdown by expression of the WT PICK1 replacement construct ascertained the specificity of the shRNA (Fig. 1*A*). In agreement with the previous report, we also found that shPICK1 was efficient in knocking down the endogenous PICK1 in primary neurons as measured through western blots (Fig. S1). In order to investigate whether knockdown of endogenous PICK1 had any effect on the surface expression of mGluR1, primary hippocampal neurons were cotransfected with myc-mGluR1 and shPICK1 or shPICK1:PICK1 (WT PICK1 replacement) constructs. Subsequently, surface myc-mGluR1 were labeled by treating live cells expressing myc-mGluR1 with anti-myc primary antibody followed by fixation of cells with 4% paraformaldehyde (PFA) and staining with goat anti-mouse Alexa-568–conjugated secondary antibody. In all our assays, surface expression of the receptors were quantified by measuring the surface fluorescence and normalizing that with the cell area (see experimental procedures section for details). The transfected cells were identified with the GFP fluorescence. The shRNA-mediated acute knockdown of endogenous PICK1 reduced the surface expression of myc-mGluR1, whereas simultaneously expressing both shPICK1 and WT PICK1 (shPICK1:PICK1) rescued the surface expression of the receptor (control: 1.0 ± 0.09; shPICK1: 0.8 ± 0.09; shPICK1:PICK1: 1 ± 0.12) (Fig. 1, *B* and *C*). These results confirmed the efficacy of the shPICK1 construct and the molecular replacement approach in primary hippocampal neurons. However, since knockdown of PICK1 resulted in decrease in the surface expression of myc-mGluR1, for studying the role of PICK1 in the agonist-mediated endocytosis of group I mGluRs, we implemented a procedure that allowed staining of both internalized and remaining surface myc-mGluR1 with different secondary antibodies and measured the proportion of surface receptors that endocytosed following ligand application (see experimental procedures section). Briefly, primary hippocampal neurons were cotransfected with myc-mGluR1 and shPICK1 or shPICK1:PICK1 constructs. Live cells expressing myc-mGluR1 were labeled with anti-myc primary antibody. Subsequent application of 100 μM R,S-3, 5-dihydroxyphenylglycine (DHPG), an agonist of group I mGluRs, resulted in the internalization of myc-mGluR1 within 30 min in control cells. This time point was selected since our earlier studies suggested that R,S-DHPG–mediated internalization of myc-mGluR1 reaches maximum level at 30 min post agonist application (13, 15). Subsequently, cells were fixed without permeabilization using ice cold 4% PFA for 15 min on ice. Surface receptors were labeled with a saturating concentration of goat anti-mouse Alexa-568–conjugated secondary antibody. Cells were then permeabilized with 0.1% Triton X-100 for 30 min at room temperature. The endocytosed receptors were then labeled by the application of goat anti-mouse Alexa-647–conjugated secondary antibody. The internalization index was then calculated by normalizing the internal fluorescence with the total fluorescence (surface + internal) as described in detail in the experimental procedures section. The transfected cells were identified with the GFP fluorescence. Importantly, acute knockdown of the endogenous PICK1 inhibited the R,S-DHPG–mediated endocytosis of myc-mGluR1, and the majority of the receptors were observed to be localized at the cell surface in shPICK1-expressing cells (control: 1 ± 0.25; control + DHPG: 1.66 ± 0.39; shPICK1 + DHPG: 1.04 ± 0.31) (Fig. 1, *D* and *E*). The inhibition of the internalization of myc-mGluR1 due to the knockdown of the endogenous PICK1 was rescued by expression of the recombinant WT PICK1 (shPICK1:PICK1 + DHPG: 1.63 ± 0.44) (Fig. 1, *D* and *E*). Thus, these results suggest that acute knockdown of endogenous PICK1 reduced the surface expression of mGluR1 and also inhibited the agonist-mediated endocytosis of the receptor in primary hippocampal neurons.

In order to investigate whether PICK1 interacts with mGluR1 and whether this interaction is dependent on the activation of the receptor by the agonist, we studied the interaction of PICK1 with mGluR1 in the absence and presence of 100 μM R,S-DHPG. Briefly, primary neurons were transfected with FLAG-mGluR1. Seventy two hours post-transfection, 100 μM R,S-DHPG was applied for 5 min. Subsequently, immunoprecipitation assay and western blots were performed following the procedure described in the experimental procedure section. PICK1 showed interaction with FLAG-mGluR1, but application of R,S-DHPG did not increase the interaction of PICK1 with the receptor (control: 1 ± 0.07; DHPG: 1.02 ± 0.07) (Fig. 1, *F* and *G*). These results are consistent with an interaction of PICK1 and mGluR1, but further experiments need to be performed to strengthen these results and to find out whether the interaction is direct or indirect between the two.

### BAR domain of PICK1 is important for the agonist-mediated internalization of mGluR1

**Figure 2:**
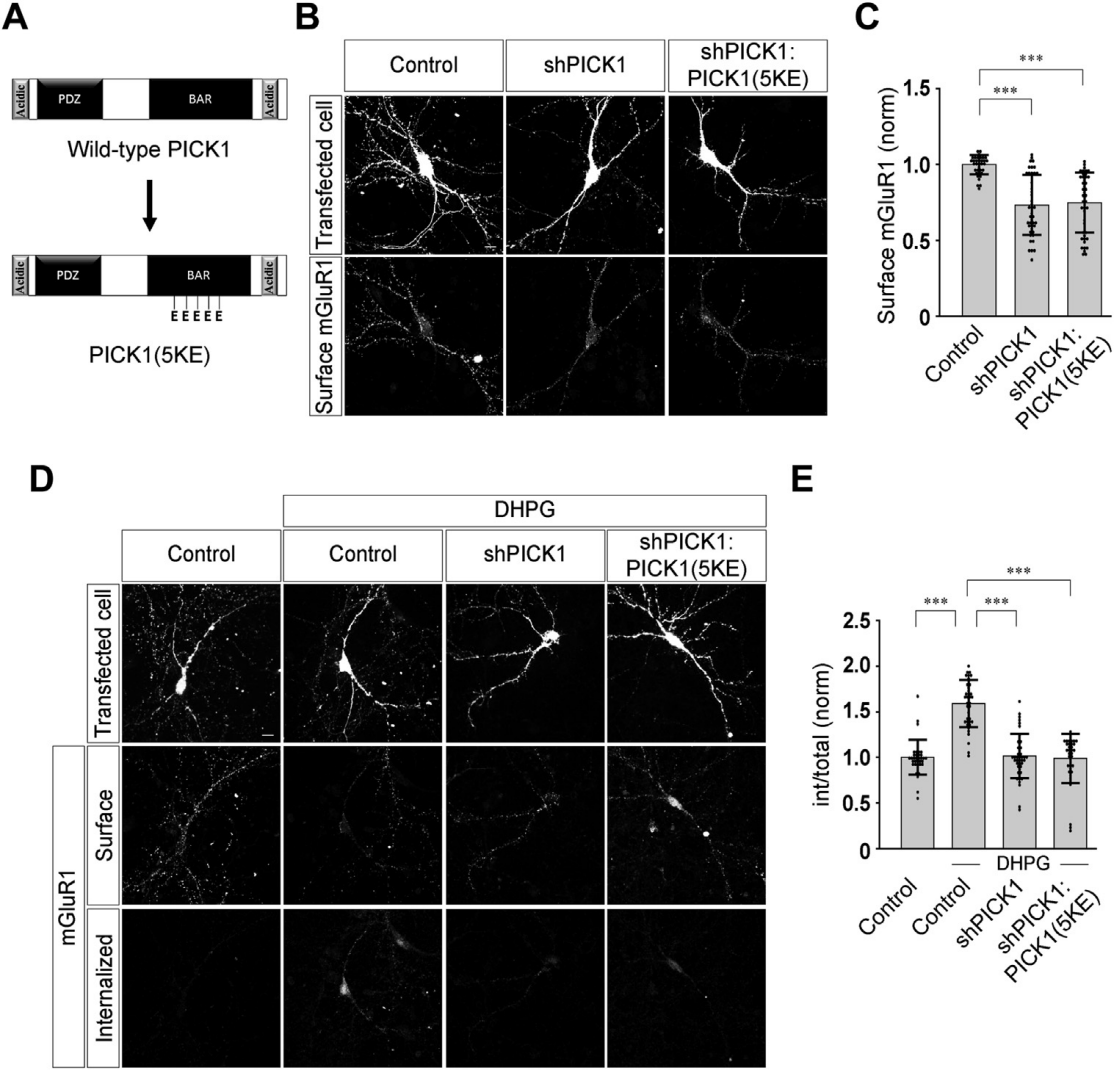
**PICK1 BAR domain plays a critical role in the surface expression and agonist-induced endocytosis of mGluR1.***A*, schematic depicting the BAR domain mutant PICK1(5KE). *B* and *C*, representative images (*B*) and quantitation (*C*) showing a decrease in the surface expression of myc-mGluR1 in shPICK1-transfected cells which could not be rescued by the expression of PICK1(5KE) replacement construct (N: control = 35; shPICK1 = 43; shPICK1:PICK1(5KE) = 43). *D* and *E*, representative images (*D*) and quantitation (*E*) suggesting that knockdown of endogenous PICK1 resulted in the inhibition of agonist-mediated endocytosis of myc-mGluR1, and expression of the PICK1(5KE) replacement construct could not rescue the endocytosis (N: control = 36; control + DHPG = 37; shPICK1 + DHPG = 42; shPICK1:PICK1(5KE) + DHPG = 37). Results are presented as means ± SD. Scale bar represents 10 μm. ∗∗∗*p* < 0.001. BAR, Bin/amphiphysin/Rvs; mGluR, metabotropic glutamate receptor; PICK1, protein interacting with C kinase 1.

PICK1 contains a BAR domain that is critical for the self-association of the protein (22). This dimerization is thought to create a large crescent shape structure through which the protein interacts with a defined membrane curvature and controls subcellular trafficking (23). This domain contains positively charged lysine residues that mediate binding to the negatively charged head groups of membrane phosphoinositides (25, 26). To investigate the function of the BAR domain of PICK1, in the agonist-mediated internalization of mGluR1, we used a replacement construct in which five lysine residues within the BAR domain were mutated to glutamic acid [shPICK1:PICK1(5KE); K251E, K252E, K257E, K266E, K268E] (Fig. 2*A*). Mutation of these residues has been shown to reduce the lipid-binding capacity, as well as the actin-binding capacity of PICK1 (24, 26). We first examined the effect of replacing endogenous PICK1 with PICK1(5KE) on the surface expression of myc-mGluR1 using the procedure described before. Knockdown of endogenous PICK1 decreased the surface expression of myc-mGluR1 (control: 1.0 ± 0.06; shPICK1: 0.73 ± 0.2) (Fig. 2, *B* and *C*). However, unlike WT PICK1, PICK1(5KE) did not rescue the surface expression of myc-mGluR1 (shPICK1:PICK1(5KE): 0.75 ± 0.2) (Fig. 2, *B* and *C*). Subsequently, we investigated the role of PICK1(5KE) in the agonist-mediated endocytosis of myc-mGluR1 using the same endocytosis assay that has been described before. In control cells, application of 100 μM R,S-DHPG increased the internalization of myc-mGluR1 at 30 min. Similar to our previous observations, shPICK1-expressing cells showed block in the myc-mGluR1 endocytosis (control: 1 ± 0.19; control + DHPG: 1.59 ± 0.26; shPICK1 + DHPG: 1.02 ± 0.24) (Fig. 2, *D* and *E*). Importantly, unlike WT PICK1, PICK1(5KE) replacement construct did not rescue the inhibition of R,S-DHPG–mediated myc-mGluR1 endocytosis caused by the knockdown of endogenous PICK1 (shPICK1:PICK1(5KE) + DHPG: 0.99 ± 0.27) (Fig. 2, *D* and *E*). These results suggest that the positively charged residues within the BAR domain of PICK1 play a critical role in the surface expression and agonist-mediated internalization of mGluR1.

### N-terminal calcium-binding region of PICK1 plays critical role in the agonist-mediated internalization of mGluR1

**Figure 3:**
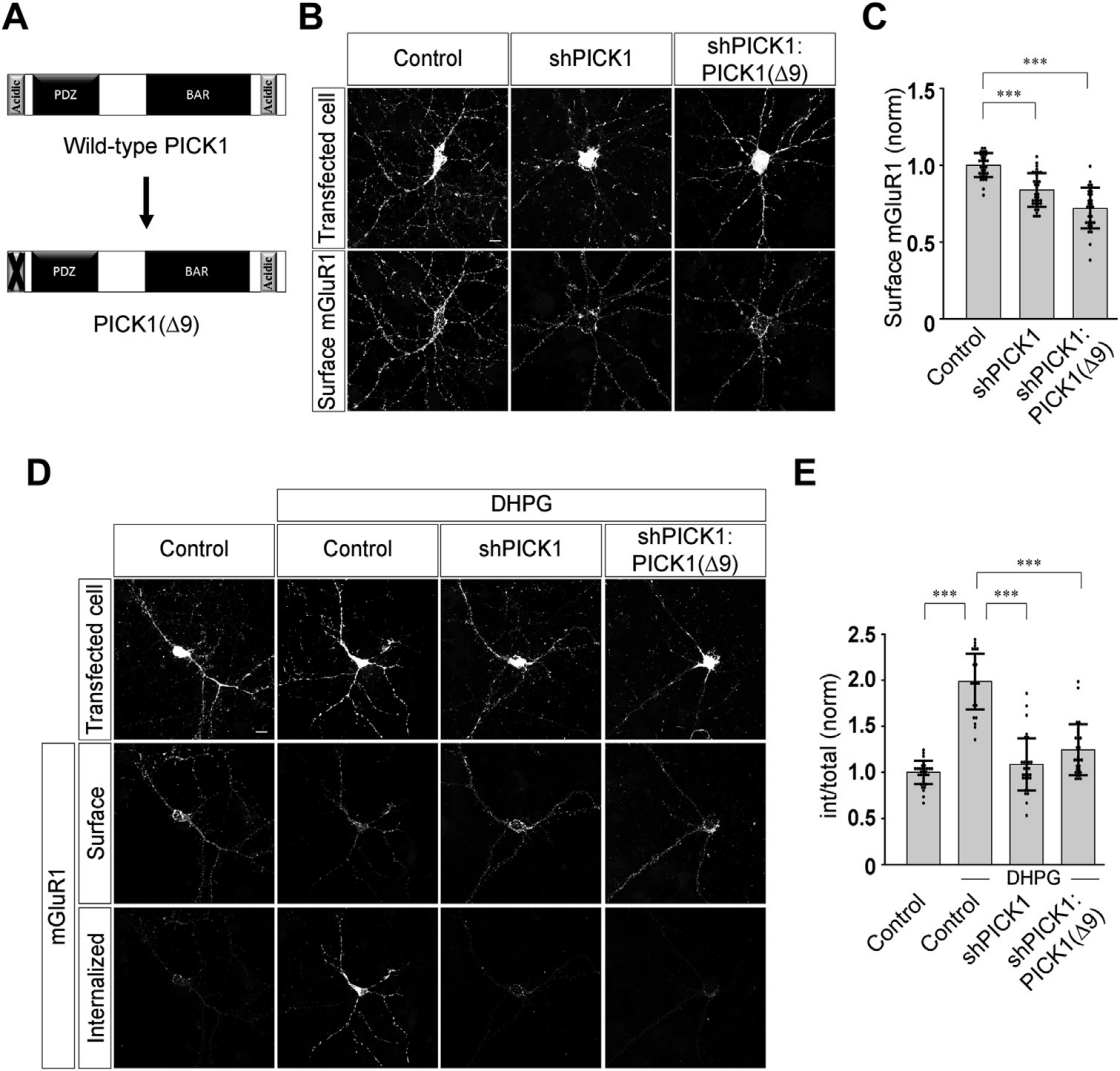
**N-terminal acidic motif of PICK1 is important for the surface expression and agonist-induced internalization of mGluR1**. *A*, schematic depicting the N-terminal domain mutant PICK1(Δ9). *B* and *C*, representative images (*B*) and quantitation (*C*) of surface myc-mGluR1 suggested that the surface localization of myc-mGluR1 decreased in shPICK1-expressing cells, and expression of PICK1(Δ9) replacement construct could not rescue the surface expression of the receptor (N: control = 32; shPICK1 = 35; shPICK1:PICK1(Δ9) = 30). *D* and *E*, representative cells (*D*) and quantitation (*E*) of 100 μM R,S-DHPG–induced internalization of myc-mGluR1 suggesting that knockdown of endogenous PICK1 led to the inhibition of agonist-mediated endocytosis of myc-mGluR1, and expression of the PICK1(Δ9) replacement construct could not rescue the normal endocytosis of the receptor (N: control = 32; control + DHPG = 29; shPICK1 + DHPG = 36; shPICK1:PICK1(Δ9) + DHPG = 30). Results are presented as means ± SD. Scale bar represents 10 μm. ∗∗∗*p* < 0.001. DHPG, 3, 5-dihydroxyphenylglycine; mGluR, metabotropic glutamate receptor; PICK1, protein interacting with C kinase 1.

Previous reports have suggested that the N-terminal acidic motif of PICK1 is important for the Ca^2+^ binding by the protein (27, 28). Ca^2+^ binding at this region promotes structural changes in PICK1 necessary for its function in regulating the AMPAR trafficking (27). In order to study the function of this calcium-binding N-terminal acidic region of PICK1 in the agonist-mediated internalization of mGluR1, we utilized a replacement construct in which the N-terminal acidic stretch of PICK1 was deleted [shPICK1:PICK1(Δ9)] (Fig. 3*A*). Expression of this replacement construct was unable to rescue the decrease in the surface myc-mGluR1 expression due to the knockdown of the endogenous PICK1, suggesting that the N-terminal acidic motif of PICK1 plays an important role in the surface expression of mGluR1 (control: 1.0 ± 0.08; shPICK1: 0.84 ± 0.11; shPICK1:PICK1(Δ9): 0.72 ± 0.13) (Fig. 3, *B* and *C*). We subsequently investigated whether this N-terminal acidic region of PICK1 is critical for the agonist-mediated endocytosis of mGluR1. As before, knockdown of endogenous PICK1 with shPICK1 inhibited the myc-mGluR1 endocytosis (control: 1 ± 0.13; control + DHPG: 1.99 ± 0.3; shPICK1 + DHPG: 1.09 ± 0.28) (Fig. 3, *D* and *E*). However, the PICK1(Δ9) replacement construct could not rescue the agonist-mediated endocytosis of myc-mGluR1 (shPICK1:PICK1(Δ9) + DHPG: 1.24 ± 0.28) (Fig. 3, *D* and *E*). These results suggest that the N-terminal acidic region of PICK1 plays a critical role in the surface localization and agonist-mediated internalization of mGluR1.

**Figure 4:**
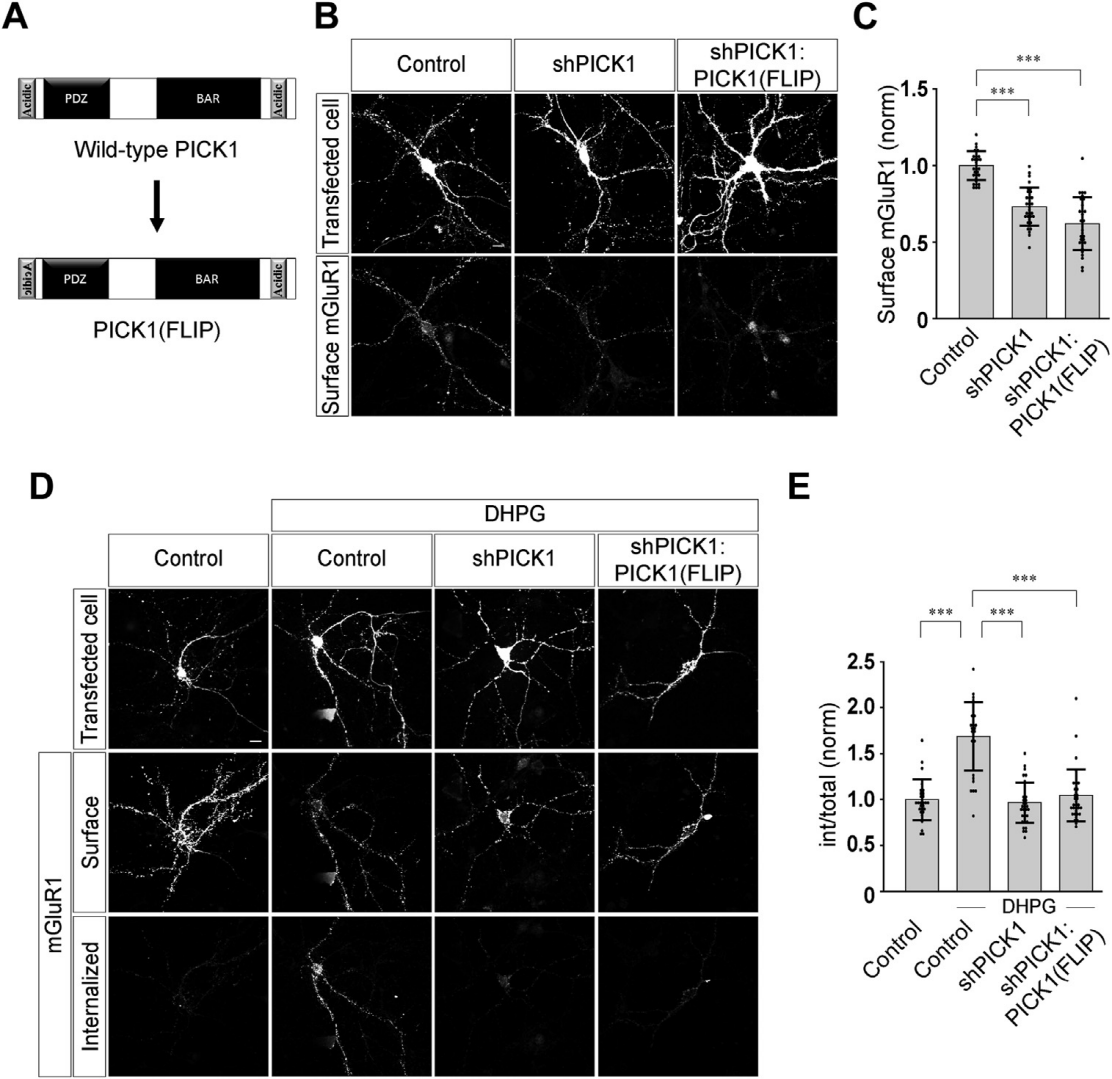
**Orientation of the N-terminal acidic motif of PICK1 is essential for the surface expression and agonist-mediated endocytosis of mGluR1.***A*, schematic depicting the N-terminal mutant PICK1(FLIP). *B* and *C*, representative images (*B*) and quantitation (*C*) of surface myc-mGluR1 in control cells and cells expressing shPICK1 or shPICK1:PICK1(FLIP) (N: control = 29; shPICK1 = 32; shPICK1:PICK1(FLIP) = 29). *D* and *E*, representative images (*D*) and quantitation (*E*) of 100 μM R,S-DHPG–mediated endocytosis of myc-mGluR1 in control cells and cells expressing shPICK1 or shPICK1:PICK1(FLIP) showing an inhibition in the endocytosis of myc-mGluR1 in both shPICK1- and shPICK1:PICK1(FLIP)-expressing cells (N: control = 29; control + DHPG = 31; shPICK1 + DHPG = 34; shPICK1:PICK1(FLIP) + DHPG = 33). Results are presented as means ± SD. Scale bar represents 10 μm. ∗∗∗*p* < 0.001. DHPG, 3, 5-dihydroxyphenylglycine; mGluR, metabotropic glutamate receptor; PICK1, protein interacting with C kinase 1.

Further, we wanted to investigate whether the total charge at the N-terminal region of PICK1 is the only important factor or the spatial arrangement of amino acids also plays a crucial role in regulating the endocytosis of mGluR1. We therefore used a PICK1 mutant in which the sequence of the N-terminal acidic motif was inverted [PICK1(FLIP)] (Fig. 4*A*). In this mutant, the total charge of the N-terminal region of PICK1 was maintained, but the spatial arrangement of amino acids was disrupted. It has been reported that inversion of this calcium-binding motif of PICK1 at the N terminus disrupts the spatial organization of amino acids necessary for calcium binding with the protein but maintains the electrostatic interactions that are required for PICK1 intramolecular folding (28). Expression of this replacement construct was unable to rescue the decrease in the surface myc-mGluR1 expression due to the knockdown of the endogenous PICK1 (control: 1.0 ± 0.09; shPICK1: 0.73 ± 0.13; shPICK1:PICK1(FLIP): 0.62 ± 0.17) (Fig. 4, *B* and *C*). Furthermore, in shPICK1:PICK1(FLIP)-transfected cells, the receptors did not endocytose upon application of 100 μM R,S-DHPG, suggesting that unlike WT PICK1, PICK1 (FLIP) could not rescue the agonist-mediated endocytosis of myc-mGluR1 (control: 1 ± 0.22; control + DHPG: 1.69 ± 0.37; shPICK1 + DHPG: 0.97 ± 0.22; shPICK1:PICK1(FLIP) + DHPG: 1.05 ± 0.28) (Fig. 4, *D* and *E*). These results suggest that not only the total charge but also the spatial arrangement of amino acids within the N-terminal acidic region of PICK1 are critical for surface expression and agonist-mediated internalization of mGluR1.

### PICK1 PDZ domain function is required for the agonist-mediated endocytosis of mGluR1

**Figure 5:**
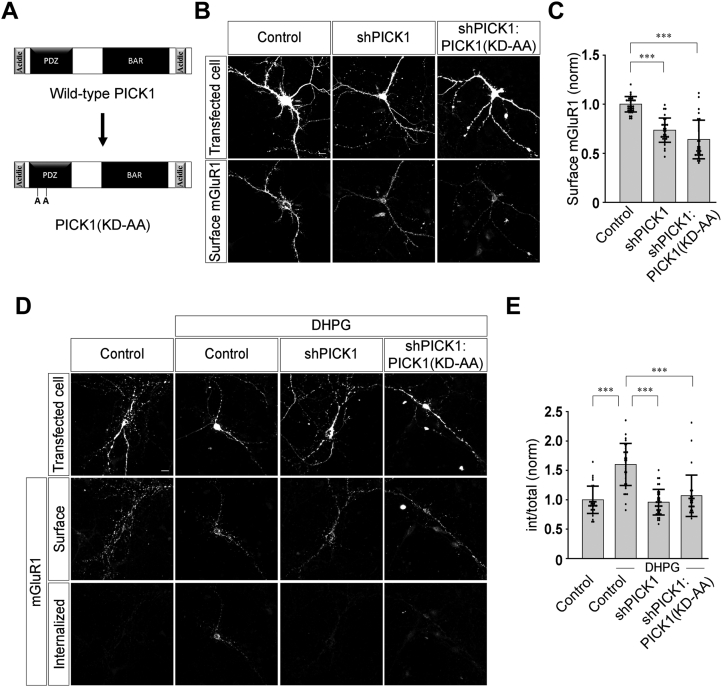
**PICK1 PDZ domain plays an important role in the surface expression and agonist-mediated endocytosis of mGluR1.***A*, schematic depicting the PDZ domain mutant PICK1(KD-AA). *B* and *C*, representative images (*B*) and quantitation (*C*) showing that knockdown of endogenous PICK1 decreases the surface expression of myc-mGluR1, and replacement of endogenous PICK1 with PICK1(KD-AA) could not rescue the surface expression of the receptor (N: control = 34; shPICK1 = 34; shPICK1:PICK1(KD-AA) = 34). *D* and *E*, representative cells (*D*) and quantitation (*E*) of the 100 μM R,S-DHPG–mediated internalization of myc-mGluR1 suggested that acute knockdown of endogenous PICK1 inhibited the endocytosis of the receptor, which could not be rescued by the expression of PICK1(KD-AA) replacement construct (N: control = 34; control + DHPG = 35; shPICK1 + DHPG = 37; shPICK1:PICK1(KD-AA) + DHPG = 31). Results are presented as means ± SD. Scale bar represents 10 μm. ∗∗∗*p* < 0.001. DHPG, 3, 5-dihydroxyphenylglycine; mGluR, metabotropic glutamate receptor; PICK1, protein interacting with C kinase 1; PDZ, PSD-95/DLG/ZO1.

A diversity of proteins of various functions has been identified as interacting with the PDZ domain of PICK1 (17, 18, 21, 34). We therefore addressed the function of the PDZ domain of PICK1, in the agonist-mediated internalization of mGluR1 using a well-characterized mutation K27A, D28A that has been reported to abolish the PICK1 PDZ domain interaction with PKC (17, 18). We used this replacement construct in which the K27 and D28 residues at the PDZ domain of PICK1 were mutated to alanine [shPICK1:PICK1(KD-AA)] (Fig. 5*A*). Initially, we studied whether the PICK1(KD-AA) replacement construct had any effect on the surface expression of myc-mGluR1 using the procedure described before. Similar to our previous results, knockdown of endogenous PICK1 reduced the surface expression of myc-mGluR1 as compared to control cells. On the other hand, expression of PICK1(KD-AA) replacement construct did not rescue the surface expression of the receptor, suggesting that these residues in the PDZ domain of PICK1 are critical for the surface expression of mGluR1 (control: 1.0 ± 0.08; shPICK1: 0.74 ± 0.12; shPICK1:PICK1(KD-AA): 0.64 ± 0.2) (Fig. 5, *B* and *C*). We subsequently investigated the effect of PICK1(KD-AA) replacement construct in the agonist-mediated endocytosis of myc-mGluR1. Knockdown of endogenous PICK1 inhibited the R,S-DHPG–mediated endocytosis of myc-mGluR1, and PICK1(KD-AA) replacement construct could not rescue this endocytosis (control: 1 ± 0.23; control + DHPG: 1.6 ± 0.36; shPICK1 + DHPG: 0.96 ± 0.22; shPICK1:PICK1(KD-AA) + DHPG: 1.07 ± 0.35) (Fig. 5, *D* and *E*). These experiments suggest that the PDZ domain of PICK1 plays a critical role in the surface expression and agonist-mediated internalization of mGluR1.

### Expression and synaptic localization of mutants of PICK1

In order to determine whether the shPICK1 and all the replacement constructs that have been used in this study effectively knocked down the endogenous PICK1, we studied the expression of endogenous PICK1 in shPICK1 and shPICK1-containing PICK1 replacement constructs expressing primary neurons using western blots. Our data suggested that all the PICK1 replacement constructs used in this study, *viz*., WT PICK1, PICK1(5KE), PICK1(Δ9), PICK1(FLIP), and PICK1(KD-AA) significantly knocked down the endogenous PICK1, and the efficiency of knockdown was similar to what was observed in cells transfected with shPICK1 alone (shPICK1:PICK1:: control: 1 ± 0.11; shPICK1: 0.5 ± 0.05; shPICK1:PICK1: 0.5 ± 0.1; shPICK1:PICK1(5KE):: control: 1 ± 0.04; shPICK1: 0.51 ± 0.02; shPICK1:PICK1(5KE): 0.44 ± 0.02; shPICK1:PICK1(Δ9):: control: 1 ± 0.1; shPICK1: 0.52 ± 0.07; shPICK1:PICK1(Δ9): 0.49 ± 0.05; shPICK1:PICK1(FLIP):: control: 1 ± 0.08; shPICK1: 0.5 ± 0.03; shPICK1:PICK1(FLIP): 0.4 ± 0.01; shPICK1:PICK1(KD-AA):: control: 1 ± 0.11; shPICK1: 0.51 ± 0.04; shPICK1:PICK1(KD-AA): 0.46 ± 0.05) (Fig. S1, *A*–*J*).

To investigate whether the PICK1 mutants that we have used in this study were targeted and localized at the synapse in primary hippocampal neurons, we studied the expression profile of each one of them. All of these constructs contained a GFP tag at the N terminus of PICK1 and produced recombinant proteins upon expression that were fused with GFP at the N terminus of the protein. As expected, our data suggested that shPICK1 effectively knocked down the endogenous PICK1. Furthermore, all the replacement constructs, that is, WT PICK1, PICK1(5KE), PICK1(Δ9), PICK1(FLIP), and PICK1(KD-AA) showed expression levels more than the endogenous PICK1, and the expression patterns of all the PICK1 constructs were very similar to the expression pattern of the WT PICK1 protein (control: 1 ± 0.85; shPICK1: 0.44 ± 0.23; shPICK1:PICK1: 4.19 ± 2.02; shPICK1:PICK1(5KE): 4 ± 1.37; shPICK1:PICK1(Δ9): 6.62 ± 2.92; shPICK1:PICK1(FLIP): 3.76 ± 1.84; shPICK1:PICK1(KD-AA): 3.62 ± 1.27) (Fig. S2, *A* and *B*). We subsequently investigated whether these mutants of PICK1 were localized to the synapse. The synapses were identified by staining for Bassoon, a core component of the active zone that is commonly used to identify synaptic terminals (35). The proportion of synapses containing detectable amount of these mutants of PICK1 were quantified by staining for clusters of GFP-PICK1 constructs or endogenous PICK1 and counterstaining for Bassoon. Our data suggested that WT PICK1, PICK1(5KE), PICK1(Δ9), PICK1(FLIP), and PICK1(KD-AA) localized at the synapse similar to the endogenous PICK1 protein (endo PICK1: 84.93 ± 9.4; wt-PICK1: 85.61 ± 11.71; PICK1(5KE): 83.76 ± 12.25; PICK1(Δ9): 84.17 ± 12.17; PICK1(FLIP): 80.31 ± 11.51; PICK1(KD-AA): 80.21 ± 15.3) (Fig. S2, *C* and *D*). It is important to point out that while all the PICK1 replacement constructs expressed to higher levels than endogenous PICK1, all of them exhibited similar levels of synaptic localization to endogenous PICK1. These results together illustrate that all PICK1 constructs used in this study expressed properly and all of them were targeted properly to the synapse, suggesting none of these manipulations in the PICK1 protein affected the expression and synaptic targeting of these constructs.

### Knockdown of PICK1 does not affect signaling by group I mGluRs and mGluR-mediated AMPAR endocytosis when both mGluR1 and mGluR5 are activated

**Figure 6:**
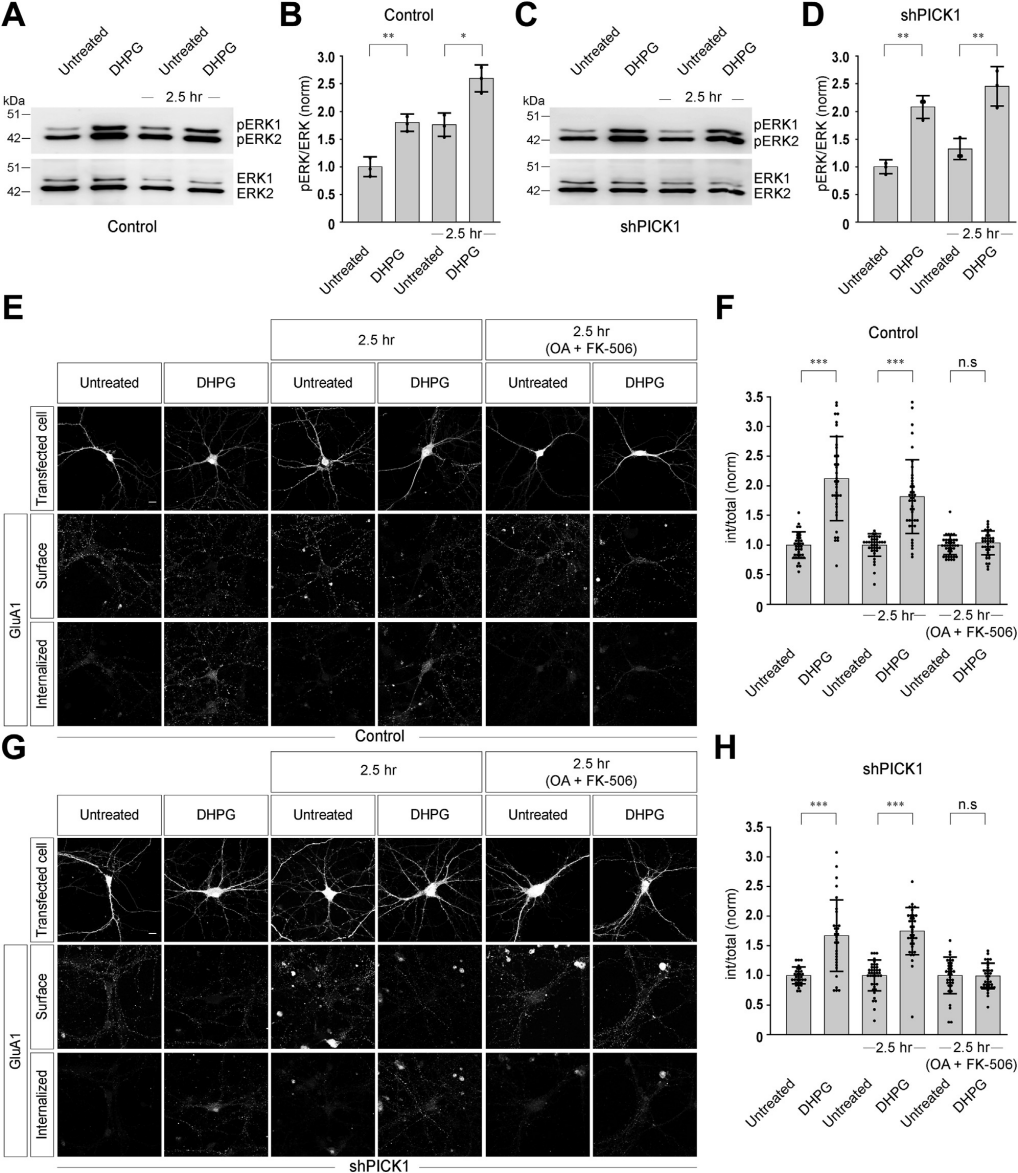
**Knockdown of endogenous PICK1 does not have any effect on mGluR-mediated signaling and AMPAR endocytosis when both mGluR1 and mGluR5 are activated.***A* and *B*, Western blot (*A*) and quantitation of the western blots (*B*) showing that in control cells, application of 100 μM R,S-DHPG for 5 min increased the phosphorylation of MAP kinases. The receptors that recycled to the cell surface in 2.5 h also induced phosphorylation of MAP kinases upon application of the agonist (N = 3). *C* and *D*, Western blot (*C*) and quantitation of the western blots (*D*) suggested that similar to control cells, in PICK1 knockdown cells, initial application of 100 μM R,S-DHPG for 5 min increased the phosphorylation of MAP kinases and application of the agonist after 2.5 h also resulted in the increase in the phosphorylation of MAP kinases (N = 3). *E* and *F*, representative images (*E*) and quantitation of the mGluR-mediated AMPAR endocytosis (*F*) suggested that in control cells, initial application of 100 μM R,S-DHPG for 5 min led to the endocytosis of AMPARs. The mGluRs that recycled back to the cell surface in 2.5 h were able to induce the endocytosis of GluA1-containing receptors when they were stimulated with 100 μM R,S-DHPG for 5 min. On the other hand, application of 100 μM R,S-DHPG did not cause endocytosis of GluA1-containing receptors when the mGluR recycling was inhibited with okadaic acid and FK-506 (N: control:: untreated = 37; DHPG = 36; 2.5 h untreated = 37; 2.5 h DHPG = 41; 2.5 h (OA + FK-506) untreated = 39; 2.5 h (OA + FK-506) DHPG = 36). *G* and *H*, representative images (*G*) and quantitation of the mGluR-mediated AMPAR endocytosis (*H*) showed that in shPICK1 transfected cells, initial application of 100 μM R,S-DHPG for 5 min led to the endocytosis of GluA1-containing receptors, and the receptors that recycled back to the cell surface in 2.5 h post agonist application also induced the internalization of GluA1-containing receptors. When the mGluR recycling was inhibited with okadaic acid and FK-506, application of 100 μM R,S-DHPG could not induce endocytosis of GluA1-containing receptors (N: shPICK1:: untreated = 31; DHPG = 31; 2.5 h untreated = 39; 2.5 h DHPG = 37; 2.5 h (OA + FK-506) untreated = 33; 2.5 h (OA + FK-506) DHPG = 35). Results are presented as means ± SD. Scale bar represents 10 μm. ∗∗∗*p* < 0.001; ∗∗*p* < 0.01; ∗*p* < 0.05; n.s, *p* > 0.05. DHPG, 3, 5-dihydroxyphenylglycine; mGluR, metabotropic glutamate receptor; PICK1, protein interacting with C kinase 1.

Subsequent to the activation by the agonist, group I mGluRs undergo desensitization and rapid internalization, followed by recycling back to the cell membrane within 2.5 h (2, 13, 14, 15). Recycling serves as a route through which these receptors are known to resensitize, that is, functionally recover, such that their responsiveness towards the agonist is restored (15). We first studied the ability of group I mGluRs to induce second messenger responses in order to investigate the effects of PICK1 knockdown on the signaling by these receptors. Group I mGluRs upregulate the phosphorylation of MAPK/ERK1/2 upon activation (15, 33, 36, 37, 38). In control cells, application of 100 μM R,S-DHPG upregulated the phosphorylation of ERK1/2. As expected, restimulation of the neurons 2.5 h following the initial stimulation further increased the phosphorylation of ERK1/2, suggesting that the recycled receptors were resensitized (control:: untreated: 1 ± 0.18; DHPG: 1.8 ± 0.16; 2.5 h:: untreated: 1.76 ± 0.21; DHPG: 2.6 ± 0.24) (Fig. 6, *A* and *B*). Interestingly, similar to control cells, in shPICK1-transfected cells, initial application of 100 μM R,S-DHPG resulted in increase in the phosphorylation of ERK1/2. Furthermore, reapplication of 100 μM R,S-DHPG after 2.5 h also resulted in increase in the group I mGluR-mediated phosphorylation of ERK1/2 (shPICK1:: untreated: 1 ± 0.13; DHPG: 2.1 ± 0.2; 2.5 h:: untreated: 1.32 ± 0.19; DHPG: 2.46 ± 0.36) (Fig. 6, *C* and *D*). These results suggest that knockdown of PICK1 does not globally affect group I mGluRs signaling when both mGluR1 and mGluR5 are activated.

We subsequently investigated the role of PICK1 on group I mGluR-mediated AMPAR trafficking. Application of various glutamate receptor agonists, including glutamate itself, NMDA, AMPA, and group I mGluR agonists, leads to the rapid endocytosis of surface AMPARs in hippocampal neurons (6, 28, 39, 40, 41). This mGluR-mediated AMPAR endocytosis is believed to be the cellular correlate of mGluR-dependent synaptic plasticity (3, 4, 37, 42). As opposed to its effect on the surface localization of group I mGluRs, knockdown of endogenous PICK1 did not affect the surface expression of GluA1-containing AMPA receptors in primary hippocampal neurons (data not shown). Since the aim of this study was to elucidate the role of PICK1 in group I mGluR internalization and in turn its effect on the mGluR-dependent AMPAR trafficking, we investigated the effect of acute knockdown of PICK1 on mGluR-mediated synaptic AMPAR endocytosis. We performed these experiments using a protocol that results in the endocytosis of synaptic AMPARs upon activation of group I mGluRs (30). In control cells, initial application of R,S-DHPG (100 μM for 5 min) resulted in the endocytosis AMPARs (control:: untreated: 1 ± 0.22; DHPG: 2.12 ± 1.04) (Fig. 6, *E* and *F*). The receptors that recycled to the cell surface in 2.5 h were also able to induce the AMPAR endocytosis when they were stimulated with 100 μM R,S-DHPG for 5 min (control:: 2.5 h untreated: 1 ± 0.19; 2.5 h DHPG: 1.83 ± 0.65) (Fig. 6, *E* and *F*). Our earlier studies suggested that the recycling of group I mGluRs depends on protein phosphatases PP2A and PP2B (13, 14). Upon blocking the recycling of the receptors by 5 nM okadaic acid and 1 μM FK-506 (PP2A and PP2B inhibitors, respectively), application of 100 μM R,S-DHPG did not induce endocytosis of AMPARs (control:: 2.5 h (OA + FK-506) untreated: 1 ± 0.16; 2.5 h (OA + FK-506) DHPG: 1.04 ± 0.2) (Fig. 6, *E* and *F*). In shPICK1-transfected cells, initial application of 100 μM R,S-DHPG for 5 min led to the endocytosis of AMPARs and application of 100 μM R,S-DHPG after 2.5 h also induced the internalization of AMPARs (shPICK1:: untreated: 1 ± 0.14; DHPG: 1.67 ± 0.6; 2.5 h untreated: 1 ± 0.26; 2.5 h DHPG: 1.75 ± 0.4) (Fig. 6, *G* and *H*). When recycling of the receptors was blocked by 5 nM okadaic acid and 1 μM FK-506, 100 μM R,S-DHPG could not induce endocytosis of AMPARs (shPICK1:: 2.5 h (OA + FK-506) untreated: 1 ± 0.31; 2.5 h (OA + FK-506) DHPG: 0.99 ± 0.22) (Fig. 6, *G* and *H*). These results suggest that the mGluR-mediated AMPAR endocytosis was normal when both mGluR1 and mGluR5 were activated by the agonist in the absence of PICK1.

### PICK1 does not play any role in the agonist-mediated endocytosis of mGluR5

**Figure 7:**
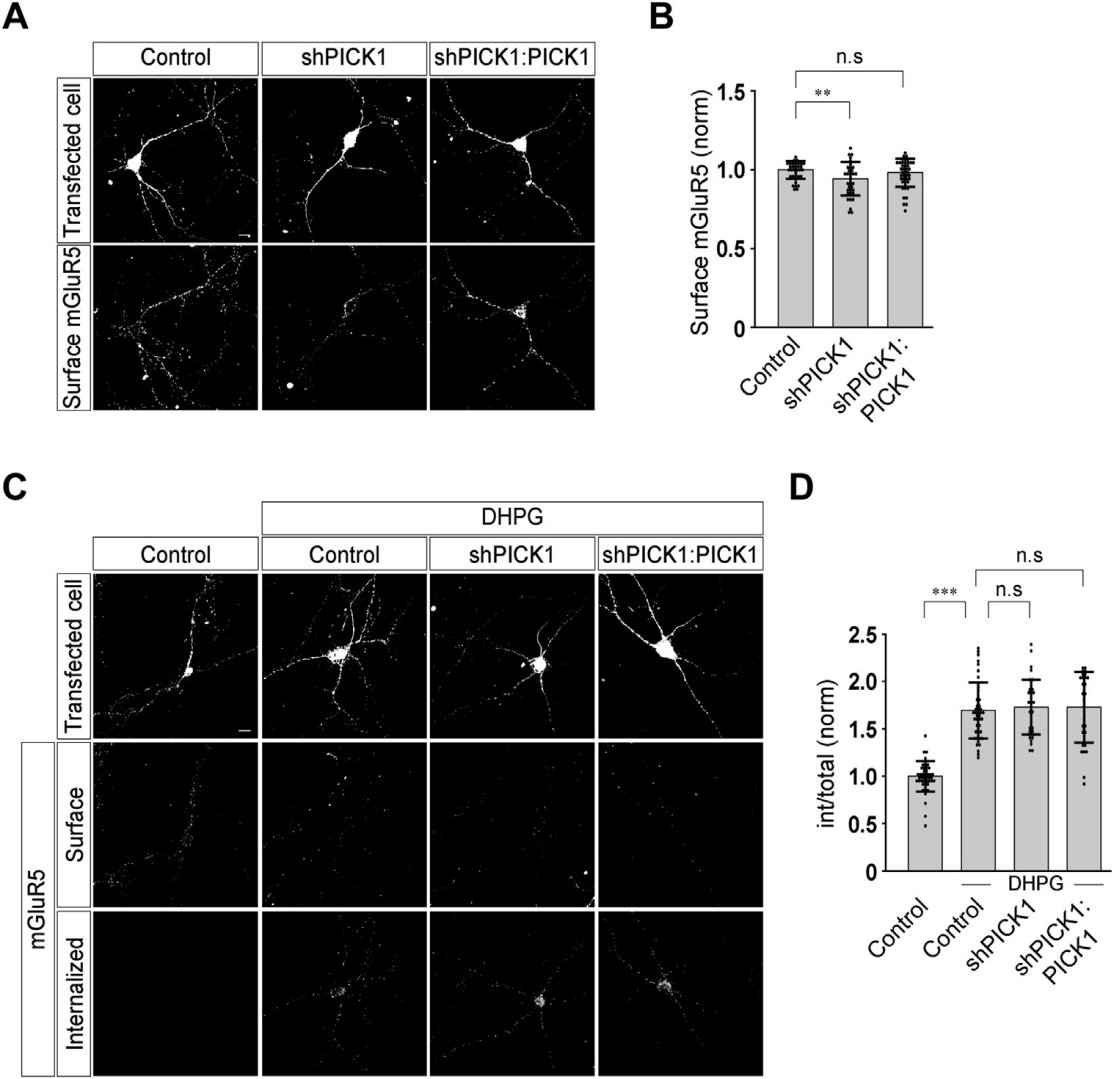
**Agonist-mediated endocytosis of mGluR5 is not affected by the knockdown of endogenous PICK1.***A* and *B*, representative images (*A*) and quantitation (*B*) of surface myc-mGluR5 suggested that following PICK1 knockdown, there was a decrease in the surface myc-mGluR5 expression that was rescued upon replacement of endogenous PICK1 with WT PICK1 (N: control = 34; shPICK1 = 33; shPICK1:PICK1 = 42). *C* and *D*, representative cells (*C*) and quantitation (*D*) of the endocytosis index suggested that knockdown of endogenous PICK1 and expression of the WT PICK1 replacement construct did not have any effect on the 100 μM R,S-DHPG–induced endocytosis of myc-mGluR5 (N: control = 45; control + DHPG = 46; shPICK1 + DHPG = 38; shPICK1:PICK1 + DHPG = 30). Results are presented as means ± SD. Scale bar represents 10 μm. ∗∗∗*p* < 0.001; ∗∗*p* < 0.01; n.s, *p* > 0.05. DHPG, 3, 5-dihydroxyphenylglycine; mGluR, metabotropic glutamate receptor; PICK1, protein interacting with C kinase 1.

Contrary to our expectations, since knockdown of endogenous PICK1 did not affect the mGluR-mediated signaling and trafficking of AMPARs when both mGluR1 and mGluR5 were activated by the agonist, we hypothesized that PICK1 probably specifically regulates the agonist-mediated internalization of mGluR1 and does not play any role in the endocytosis of the other member of the group I mGluR family, mGluR5. We therefore studied the role of PICK1 in the agonist-mediated endocytosis of mGluR5 using the procedure described in the experimental procedures section. Knockdown of endogenous PICK1 slightly reduced the surface expression of myc-mGluR5 that was rescued by expression of the WT PICK1 (control: 1.0 ± 0.06; shPICK1: 0.94 ± 0.11; shPICK1:PICK1: 0.98 ± 0.09) (Fig. 7, *A* and *B*). Interestingly, knockdown of the endogenous PICK1 and replacement of the endogenous PICK1 with WT PICK1 had no effect on the 100 μM R,S-DHPG–mediated internalization of myc-mGluR5 (control: 1 ± 0.16; control + DHPG: 1.69 ± 0.3; shPICK1 + DHPG: 1.73 ± 0.29; shPICK1:PICK1 + DHPG: 1.73 ± 0.37) (Fig. 7, *C* and *D*). Thus, these results suggest that although PICK1 plays a role in the surface stabilization of mGluR5, it does not play any role in the agonist-dependent internalization of mGluR5.

### PICK1 specifically regulates the mGluR1-mediated signaling and mGluR1-mediated AMPAR trafficking

**Figure 8:**
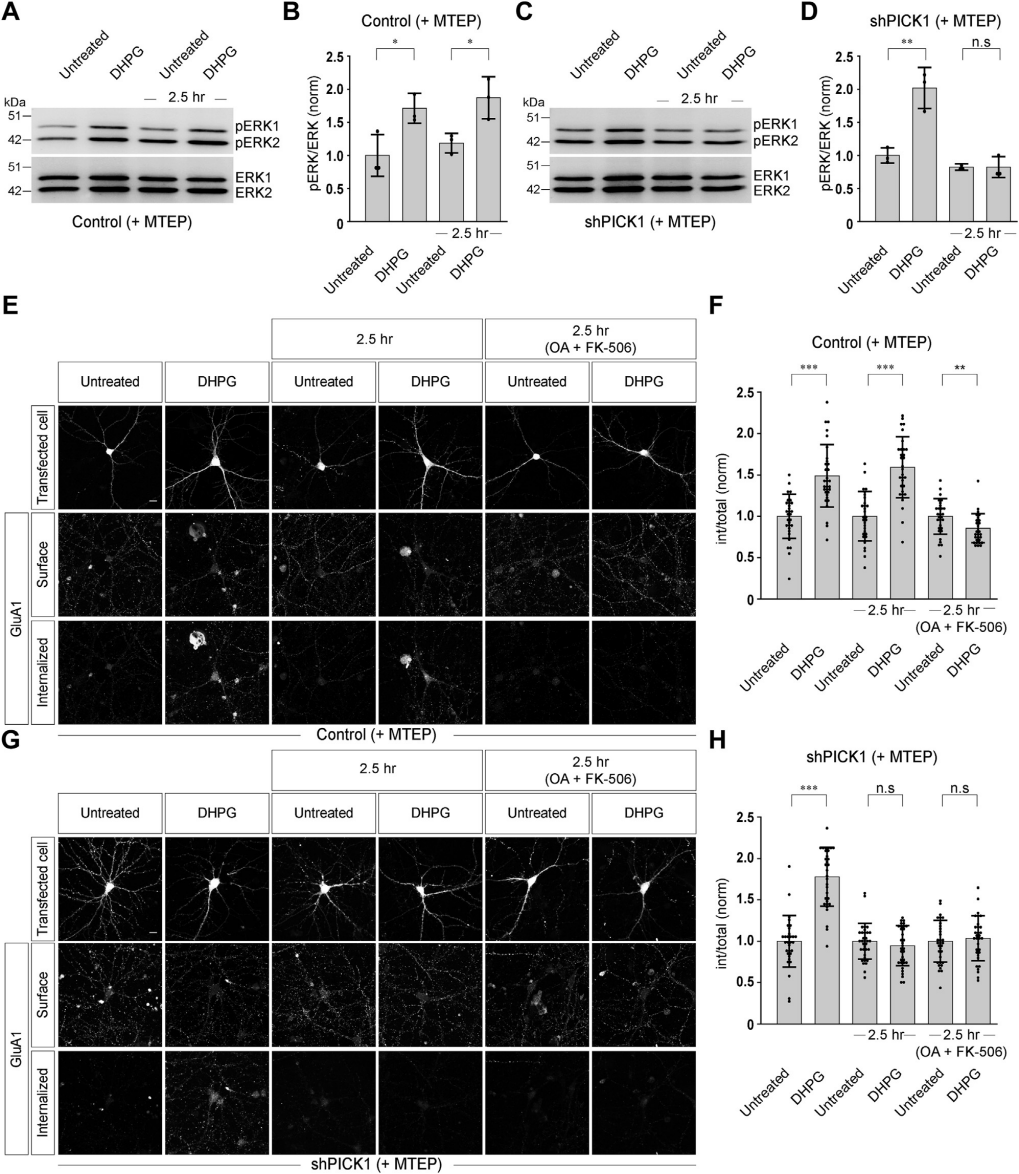
**PICK1 is specifically involved in the mGluR1-mediated signaling and mGluR1-mediated AMPAR endocytosis.***A* and *B*, Western blot (*A*) and quantitation of the western blots (*B*) showing that in control cells that were treated with the mGluR5 antagonist MTEP, application of 100 μM R,S-DHPG for 5 min led to the phosphorylation of MAP kinases. Furthermore, the receptors recycled to the cell surface in 2.5 h and also showed the ability to induce phosphorylation of MAP kinases on application of 100 μM R,S-DHPG for 5 min (N = 3). *C* and *D*, Western blot (*C*) and quantitation of the western blots (*D*) suggested that although in PICK1 knockdown cells, in presence of MTEP, initial application of 100 μM R,S-DHPG for 5 min led to the phosphorylation of MAP kinases, but application of 100 μM R,S-DHPG after 2.5 h did not increase the phosphorylation of MAP kinases (N = 3). *E* and *F*, representative images (*E*) and quantitation of the endocytosis index (*F*) suggested that in control cells (treated with MTEP), the receptors that recycled back to the cell surface in 2.5 h were able to induce the internalization of GluA1-containing receptors when they were stimulated with 100 μM R,S-DHPG for 5 min. Application of 100 μM R,S-DHPG did not cause endocytosis of GluA1-containing receptors when the mGluR1 recycling was inhibited with okadaic acid and FK-506 (N: control:: untreated = 30; DHPG = 32; 2.5 h untreated = 30; 2.5 h DHPG = 31; 2.5 h (OA + FK-506) untreated = 29; 2.5 h (OA + FK-506) DHPG = 29). *G* and *H*, representative images (*G*) and quantitation of the mGluR-mediated AMPAR endocytosis (*H*) showed that in shPICK1 transfected cells that were treated with mGluR5 antagonist MTEP, although initial application of 100 μM R,S-DHPG for 5 min led to the endocytosis of GluA1-containing receptors, application of 100 μM R,S-DHPG after 2.5 h did not induce the internalization of GluA1-containing receptors. Expectedly, in okadaic acid and FK-506–treated cells, 100 μM R,S-DHPG did not cause endocytosis of GluA1-containing receptors (N: shPICK1:: untreated = 31; DHPG = 32; 2.5 h untreated = 34; 2.5 h DHPG = 33; 2.5 h (OA + FK-506) untreated = 33; 2.5 h (OA + FK-506) DHPG = 32). Results are presented as means ± SD. Scale bar represents 10 μm. ∗∗∗*p* < 0.001; ∗∗*p* < 0.01; ∗*p* < 0.05; n.s, *p* > 0.05. DHPG, 3, 5-dihydroxyphenylglycine; mGluR, metabotropic glutamate receptor; PICK1, protein interacting with C kinase 1; MTEP, (3-((2-methyl-1,3-thiazol-4-yl) ethynyl) pyridine).

Our earlier results suggested that PICK1 showed contrasting effects in case of agonist-mediated internalization of mGluR1 and mGluR5. Since knockdown of endogenous PICK1 did not affect the mGluR-mediated signaling and trafficking of AMPARs when both mGluR1 and mGluR5 were activated by the agonist, we hypothesized that PICK1 probably specifically regulates the mGluR1-mediated signaling and AMPAR trafficking and in the absence of PICK1, mGluR5-mediated signaling and AMPAR trafficking remain normal. In order to test our hypothesis, we first investigated the ability of mGluR1 alone to induce the second messenger responses upon binding with the agonist in control cells and PICK1 knockdown cells by performing the experiments using the method described above in the presence of 100 μM (3-((2-methyl-1,3-thiazol-4-yl) ethynyl) pyridine) (MTEP), a specific antagonist of mGluR5. In control cells, activation of mGluR1 by 100 μM R,S-DHPG upregulated the phosphorylation of ERK1/2 and the receptors that recycled to the cell surface in 2.5 h also increased the phosphorylation of ERK1/2 upon activation by 100 μM R,S-DHPG (control:: untreated: 1 ± 0.32; DHPG: 1.71 ± 0.22; 2.5 h:: untreated: 1.19 ± 0.15; DHPG: 1.87 ± 0.32) (Fig. 8, *A* and *B*). Importantly, in shPICK1-transfected cells, although initial application of 100 μM R,S-DHPG increased the phosphorylation of ERK1/2, application of 100 μM R,S-DHPG after 2.5 h did not increase the mGluR1-mediated phosphorylation of ERK1/2 (shPICK1:: untreated: 1 ± 0.12; DHPG: 2.02 ± 0.31; 2.5 h:: untreated: 0.82 ± 0.05; DHPG: 0.82 ± 0.16) (Fig. 8, *C* and *D*). These results suggest that in the absence of PICK1, mGluR1 could not internalize subsequent to the activation by the agonist and therefore could not resensitize, retaining the receptor in an inactive form on the cell membrane.

We subsequently investigated the role of PICK1 in mGluR1-mediated AMPAR trafficking in control cells and shPICK1-expressing cells using the method described above after blocking mGluR5 by MTEP. In control cells, AMPARs endocytosed after the initial application of 100 μM R,S-DHPG for 5 min and receptors that recycled back to the cell surface in 2.5 h were also able to induce the endocytosis of AMPARs (control:: untreated: 1 ± 0.27; DHPG: 1.49 ± 0.38; 2.5 h untreated: 1 ± 0.3; 2.5 h DHPG: 1.59 ± 0.37) (Fig. 8, *E* and *F*). When the receptors were unable to recycle in the presence of 5 nM okadaic acid and 1 μM FK-506, 100 μM R,S-DHPG did not induce endocytosis of AMPARs (control:: 2.5 h (OA + FK-506) untreated: 1 ± 0.22; 2.5 h (OA + FK-506) DHPG: 0.86 ± 0.18) (Fig. 8, *E* and *F*). On the other hand, in shPICK1-transfected cells, although initial application of 100 μM R,S-DHPG for 5 min led to the endocytosis of AMPARs, application of 100 μM R,S-DHPG after 2.5 h did not induce the internalization of AMPARs (shPICK1:: untreated: 1 ± 0.31; DHPG: 1.78 ± 0.36; 2.5 h untreated: 1 ± 0.22; 2.5 h DHPG: 0.95 ± 0.24) (Fig. 8, *G* and *H*). As expected, 5 nM okadaic acid 1 μM FK-506–treated cells also did not show AMPAR endocytosis upon application of 100 μM R,S-DHPG (shPICK1:: 2.5 h (OA + FK-506) untreated: 1 ± 0.25; 2.5 h (OA + FK-506) DHPG: 1.03 ± 0.27) (Fig. 8, *G* and *H*). These results suggest that PICK1 plays critical role in the internalization and resensitization of mGluR1 and it specifically regulates the mGluR1-mediated AMPAR endocytosis. PICK1 does not play any role in the mGluR5-mediated internalization of AMPARs.

## Discussion

Trafficking plays a crucial role in controlling the localization of group I mGluRs in a specific region of the neuron and also regulates the activity of the receptors. Improper trafficking of the receptors could result in abnormal signaling, which might have serious pathological consequences. Due to these reasons, understanding the detailed molecular mechanisms underlying the trafficking of group I mGluRs has been of great interest. In the present study, we have defined a novel role for PICK1 in the agonist-mediated internalization of group I mGluRs and mGluR-mediated AMPAR endocytosis, which is believed to be the cellular correlate for mGluR-dependent synaptic plasticity. PICK1 contains an N-terminal PDZ domain that was originally found to bind the extreme C terminus of PKC_α_ but was later shown to also bind the C termini of several other proteins (17, 18, 19, 21, 34). Through its lipid-binding BAR domain, PICK1 targets its binding partners to the inner surface of the cell, thereby influencing their synaptic localization and function (23). The regulation of PICK1 and some PICK1-interacting partners seems to be altered in certain pathological conditions, suggesting the involvement of PICK1 in neurological disorders. On the basis of a series of mutant PICK1 constructs, primarily using a strategy in which mutant forms of PICK1 replace endogenous PICK1 that has been knocked down by shRNA, we studied the role of PICK1 in the surface expression and agonist-mediated internalization of group I mGluRs. We have demonstrated that although PICK1 plays an important role in the surface stabilization of both members of the group I mGluR family, mGluR1 and mGluR5, it specifically regulates the agonist-mediated endocytosis of mGluR1, while mGluR5 internalization by the agonist remains unaffected in the absence of PICK1. Thus, PICK1 affected the surface expression of mGluR1 and mGluR5 independent of its effects on their endocytosis. Our data suggest that PICK1 probably interacts with mGluR1, but further experiments are needed to be performed to strengthen this conclusion and to find out whether the interaction between the two is direct or indirect. Agonist-mediated internalization of group I mGluRs can be regulated by various scaffolding proteins that form a dense network within the PSD. We had earlier established a key role for three scaffolding proteins, tamalin, Norbin, and sorting nexin 1, in the agonist-mediated endocytosis and recycling of group I mGluRs (15, 32, 33). All these proteins regulated the agonist-mediated trafficking of both members of the group I mGluR family, mGluR1 and mGluR5. PICK1 is the first protein that showed differential regulation in the agonist-mediated internalization of mGluR1 and mGluR5; it specifically regulated the agonist-mediated endocytosis of mGluR1, but not mGluR5. For example, tamalin regulates the trafficking of both the members of group I mGluR family through its interaction with S-SCAM (32). We have also shown that another PSD protein Norbin acts as a dual regulator of both mGluR trafficking as well as mGluR-mediated AMPAR endocytosis through its interaction with PKA. The other postsynaptic protein sorting nexin 1, together with Hrs, plays a crucial role in the recycling of group I mGluRs through the “slow” recycling route which is important for the “resensitization” of these receptors (15). Although it is currently unknown how these postsynaptic proteins collaborate with each other to regulate the group I mGluR trafficking and mGluR-mediated AMPAR endocytosis, our findings suggest that the large network of postsynaptic scaffolding proteins beneath the postsynaptic region plays a very important role in the trafficking of these receptors, hence maintaining the homeostasis and spatiotemporal localization of these receptors.

We found that the BAR domain of PICK1 had an important role to play in the surface expression and agonist-mediated internalization of mGluR1. The PICK1(5KE) replacement construct in which five lysine residues within the BAR domain of PICK1 were mutated to glutamic acid could not rescue the surface expression and agonist-mediated endocytosis of mGluR1. This replacement construct has been reported to disrupt the interaction between positive charges on the concave side of the crescent-shaped BAR domain of PICK1 and negative charges on the lipid head groups of the membrane, suggesting that this interaction is important for surface expression as well as agonist-mediated internalization of mGluR1 (23, 25, 26). In order to check the function of calcium-binding N-terminal acidic region of PICK1 in mGluR1 internalization, we used a replacement construct in which the extreme nine amino acids at the N terminus of PICK1 were deleted [PICK1(Δ9)]. This PICK1(Δ9) replacement construct could not rescue the surface expression as well as agonist-mediated endocytosis of mGluR1. Reports have suggested that calcium binding to the N-terminal region promotes structural changes in PICK1 (27, 28). Therefore, it is possible that calcium binding to the N-terminus region of PICK1 promotes conformational changes in PICK1 that is critical for its function in regulating the surface expression as well as agonist-mediated endocytosis of mGluR1. This hypothesis needs to be investigated in future. We next used a replacement construct in which the acidic stretch of the N-terminus region of PICK1 was flipped [PICK1(FLIP)] in order to determine whether charge in this region is the only important factor or the arrangement of the amino acids also plays a critical role in the agonist-mediated internalization of mGluR1. The inability of PICK1(FLIP) replacement construct to rescue the normal endocytosis of mGluR1 pointed out that not only the charge but the spatial arrangement of amino acids at this region is also important for calcium binding which further affects the mGluR1 internalization. The PDZ domain of PICK1 interacts with many proteins; most of them are membrane proteins, receptors, transporters, and ion channels (16, 18, 21, 34). It has been reported that mutation of K27 together with D28 to AA completely abolishes the PICK1 PDZ domain interaction with PKC (17, 28). Our data suggest that this mutation inhibits the agonist-mediated internalization and also reduces the surface expression of mGluR1. This may be because PICK1 facilitates the phosphorylation of its substrates by recruiting protein kinase C_α_, which further regulates the trafficking of mGluR1. This hypothesis also needs to be investigated in future.

We finally studied the role of PICK1 in the mGluR-mediated signaling and mGluR-dependent AMPAR endocytosis, which is a prerequisite for the mGluR-mediated synaptic plasticity. Interestingly, unlike its effect on mGluR surface expression, knockdown of PICK1 had no effect on the surface expression of GluA1-containing receptors, suggesting that its effect on the surface localization is specific for group I mGluRs. Importantly, we show here that mGluR1 that could not internalize in the absence of PICK1 did not get resensitized and were inactive. In other words, those receptors were unable to induce the second messenger responses as observed by their inability to upregulate the phosphorylation of MAP kinases. Moreover, they were also unable to induce the AMPAR endocytosis. On the other hand, when the receptors were allowed to recycle back to the cell surface in control cells, the recycled receptors were able to upregulate the phosphorylation of MAP kinases and induce the AMPAR endocytosis. These results indicate that PICK1-mediated internalization of mGluR1 is critical for the resensitization of these receptors. Our results also suggested that the effect of PICK1 was specific for mGluR1-mediated processes. mGluR5-mediated MAPK signaling and AMPAR endocytosis were not dependent on PICK1. An earlier report suggested that knockdown of PICK1 had no effect on the mGluR-mediated AMPAR endocytosis (28). It is worth pointing out that in that study, AMPAR endocytosis was induced by the activation of both mGluR1 and mGluR5. As mentioned before, PICK1 specifically regulates mGluR1 internalization and does not have any effect on mGluR5. Therefore, effect of PICK1 was observed when the AMPAR endocytosis was studied by specific activation of mGluR1 after blocking mGluR5. It is known that in various parts of the brain including hippocampus, mGluR1 and mGluR5 have different physiological roles. This study clearly indicates that the two subtypes of group I mGluRs, mGluR1 and mGluR5, probably use distinct and separate intracellular mechanisms to mediate different cellular effects. Our study also suggests that in the hippocampal neurons, mutation in the PICK1 protein would probably affect the mGluR1-mediated processes but mGluR5-mediated cellular processes would be unaffected. Thus, this study provides a novel mechanism for the regulation of agonist-mediated internalization of mGluR1 and mGluR1-mediated AMPAR trafficking by PICK1.

As stated before, inappropriate trafficking of group I mGluRs could lead to improper signaling with pathological consequences. Inappropriate signaling of group I mGluRs has been suggested to be involved in the pathophysiology of multiple cognitive disorders such as fragile X syndrome, autism etc (3, 7, 8, 9). Indeed, our data suggest that in the absence of PICK1, subsequent to the activation by the agonist, mGluR1 stays at the cell surface in an inactive form and is unable to participate in mGluR1-mediated synaptic plasticity. Therefore, the PICK1-mediated internalization of these receptors appears to be critical for the resensitization of the receptor. Thus, our results reveal a crucial role of PICK1 in controlling the normal trafficking of mGluR1, failure of which could lead to impaired mGluR1-mediated synaptic plasticity, and it can have clinical relevance to the function of mGluR1 in the above mentioned neuropsychiatric disorders.

## Experimental procedures

### Materials

Dulbecco's Modified Eagle Medium, fetal bovine serum, Minimal Essential Medium, Neurobasal medium, B27 supplement, antibiotic-antimycotic mix, Trypsin-EDTA, and other reagents for cell culture were purchased from Invitrogen. All salts and chemicals like fluorodeoxyuridine, poly-D-lysine, PFA, PEI, and Fluoromount aqueous mounting medium were purchased from Sigma. FemtoLUCENT plus-HRP kit was purchased from G-Biosciences. Protein A/G PLUS agarose beads were purchased from Santa Cruz Biotechnology, and femtoLUCENT plus-HRP kit was purchased from G-Biosciences. R,S-DHPG, APV, and DNQX (6,7-dinitroquinoxaline-2,3-dione) were obtained from Tocris. Tetrodotoxin citrate was purchased from Abcam. MTEP was purchased from Sigma. Fine chemicals were obtained from Life technologies and Merck limited. Anti-myc mouse monoclonal and anti-PICK1 goat polyclonal antibodies were purchased from Abcam. Anti-HA rat monoclonal antibody was from Roche, and anti-GFP rabbit polyclonal antibody was from Life technologies. Anti-FLAG rabbit polyclonal antibody was from Sigma. Anti-Bassoon mouse monoclonal antibody was purchased from Enzo Life Sciences. ERK1/2 mouse monoclonal and phospho-ERK1/2 rabbit monoclonal antibodies were purchased from Cell Signaling Technology. Anti-GluA1 rabbit polyclonal antibody was purchased from Millipore. Anti-β actin antibody was obtained from Santa Cruz Biotechnology. Alexa Fluor–conjugated secondary antibodies were purchased from Invitrogen, and horseradish peroxidase (HRP)-conjugated secondary antibodies were purchased from Sigma. ECL Western blot detection kit was obtained from GE Healthcare.

### Constructs

In myc-mGluR1 and myc-mGluR5 constructs, the myc epitope was tagged at the N terminus of the full-length mGluR1 and mGluR5. These constructs were used in earlier studies (13, 14, 15, 30, 32, 33, 43). The FLAG-mGluR1 construct was obtained from Johanna Montgomery’s lab (University of Auckland). All the constructs of PICK1 that have been used in this study were previously used in another study (28). Briefly, the shRNA against PICK1 (shPICK1) was cloned in a multipromoter vector under the H1 promoter targeting the PICK1 sequence. This shRNA was found to be very effective in knocking down the endogenous PICK1 in primary neurons (28). PICK1 replacement constructs were cloned under the ubiquitin promoter of the vector containing shPICK1. All the replacement constructs *viz*., shPICK1:PICK1 (full-length PICK1), shPICK1:PICK1(5KE) (five lysine residues within the BAR domain of PICK1 were mutated to glutamic acid (K251E, K252E, K257E, K266E, K268E)), shPICK1:PICK1(Δ9) (extreme nine amino acids at the N terminus of PICK1 were deleted), shPICK1:PICK1(FLIP) (the sequence of the N-terminal acidic motif of PICK1 was inverted), shPICK1:PICK1(KD-AA) (K27 and D28 residues of PICK1 were mutated to alanine) were tagged with HA epitope and GFP at the N terminus of the protein. The expression pattern of the N-terminally GFP-tagged PICK1 constructs used in this study has been reported to be similar to that of the endogenous protein (28). All replacement constructs described above were based on an shRNA-resistant version of PICK1, in which silent mutations were introduced in the shPICK1 target region of PICK1, preventing knockdown of the replacement constructs by shPICK1.

### Dissociated hippocampal neuron cultures and transfection

Primary hippocampal neuron cultures were prepared from P0 C57BL/6 mouse pups, as described previously with minor changes (33). Briefly, hippocampi were dissected out from P0 mouse pups. Subsequently, tissue was dissociated by papain treatment. Cells were then plated on coverslips coated with poly-D-lysine in sodium borate solution (50 μg/ml poly-D-lysine + 0.1 M sodium borate) at a density of approximately 150,000 cells per 12 mm well in a 24-well plate. Cultures were maintained in neurobasal media with B27 supplement and 0.5 mM glutamine. Fluorodeoxyuridine was added on the fourth day of culture to stop the glial growth. All the studies were approved by the Institutional Animal Ethics Committee of Indian Institute of Science Education and Research Mohali.

Primary hippocampal neurons were transfected with myc-mGluR1/myc-mGluR5, shPICK1, and various PICK1 replacement constructs at 7 to 8 days *in vitro* (DIV) using calcium phosphate method (33, 44). All the experiments were performed when the cells were at 12 to 14 DIV.

### Surface receptor staining assay

In order to investigate the effect of knockdown of endogenous PICK1 and expression of various replacement constructs of PICK1 on the surface expression of group I mGluRs, primary hippocampal neurons were cotransfected with myc-mGluR1/myc-mGluR5 and shPICK1 or shPICK1-containing PICK1 replacement constructs. Four to five days after transfection, surface myc-mGluR1/myc-mGluR5 was labeled with mouse anti-myc primary antibody (1:200) for 20 min at 37 °C in live cells, followed by fixation in 4% PFA. Subsequently, surface receptors were labeled by the application of goat anti-mouse Alexa-568–conjugated secondary antibody (1:100) for 1 h at 37 °C. Cells transfected with shPICK1 or GFP, and HA-tagged PICK1 replacement constructs were stained with anti-GFP (1:500) and anti-HA primary antibodies (1:500) respectively for overnight at 4 °C, followed by staining with appropriate Alexa-488–conjugated secondary antibodies. The coverslips were then mounted on glass slides and imaged under the confocal microscope.

### Endocytosis assay

Primary hippocampal neurons were cotransfected with myc-mGluR1/myc-mGluR5 complementary DNA and with either shPICK1 or shPICK1-containing PICK1 replacement constructs as described above. Experiments were performed 5 to 7 days after transfection. Live cells expressing myc-mGluR1/myc-mGluR5 were treated with mouse anti-myc primary antibody (1:200) for 20 min at 37 °C. Subsequently, cells were treated with 100 μM R,S-DHPG, which is a specific agonist of group I mGluRs, for 5 min. Cells were then chased at 37 °C in plain neurobasal media in the absence of the agonist for various time periods. Following that, cells were fixed without permeabilization using ice cold 4% PFA for 15 min on ice. Surface-localized receptors were then labeled with the saturating concentration of goat anti-mouse Alexa-568–conjugated secondary antibody (1:100) for 1 h at 37 °C, followed by permeabilization of the cells with 0.1% Triton X-100 for 30 min at room temperature. The endocytosed receptors were then labeled by the application of goat anti-mouse Alexa-647–conjugated secondary antibody (1:700) for 1 h at 37 °C. Cells transfected with shPICK1 or other replacement constructs of PICK1 tagged with GFP and HA were stained with anti-GFP (1:500) and anti-HA primary antibodies (1:500), respectively, for overnight at 4 °C. After that, they were stained with appropriate Alexa-488–conjugated secondary antibodies. The coverslips were then mounted on glass slides and imaged under the confocal microscope. In order to ensure that the Alexa-647–conjugated secondary antibody that we used to detect internalized receptors did not label any detectable surface receptors in our assays, we performed control experiments to determine the saturating concentration of the first secondary antibody similar to what we have described in our earlier studies (30, 33). The control experiments suggested that in our assays, Alexa-647–conjugated secondary antibody did not label any detectable amount of surface receptors and thus it stained the internalized receptors only (data not shown).

To investigate the role of PICK1 in the mGluR-mediated AMPAR endocytosis, primary hippocampal neurons were transfected with shPICK1 or WT PICK1 replacement construct as described above. Subsequently, the mGluR-mediated AMPAR endocytosis assay was performed in the presence or absence of mGluR5 antagonist, MTEP, using the protocol used before (33). Briefly, to study the role of both members of group I mGluR family, mGluR1 and mGluR5 in the AMPAR endocytosis (in the presence and absence of endogenous PICK1), cells were preincubated in 1 μM tetrodotoxin citrate (presynaptic release blocker), 20 μM DNQX (AMPAR antagonist), and 50 μM APV (NMDAR antagonist) for 30 min at 37 °C. On the other hand, to study the role of only mGluR1 in the AMPAR endocytosis (in the presence and absence of endogenous PICK1), cells were preincubated with mGluR5 antagonist, MTEP (100 μM), in addition to the other blockers as mentioned above for 30 min at 37 °C. Subsequently, in control cells, internalization of group I mGluRs was induced by the application of 100 μM R,S-DHPG for 5 min. Cells were then chased in the absence of the agonist, and mGluRs were allowed to recycle back to the cell surface in 2.5 h. In another set of control cells, the recycling of mGluRs was inhibited by the application of 5 nM okadaic acid and 1 μM FK-506. In shPICK1-transfected cells, 100 μM R,S-DHPG was applied for 5 min to activate the receptors and cells were chased for 2.5 h in the absence of the agonist. Subsequent to the appropriate recycling time of the receptors, GluA1-containing receptors were labeled in live neurons by 15 min incubation at 37 °C with a rabbit polyclonal antibody directed against the N terminus of the GluA1 subunit (1:150). To measure the AMPAR endocytosis induced by the mGluRs, when they were initially present at the cell surface in both control cells and shPICK1-transfected cells, cells were treated with appropriate antagonists and GluA1-containing receptors were labeled with the antibody in live cells as described above. In all these conditions, subsequent to the labeling of GluA1-containing receptors, 100 μM R,S-DHPG was applied for 5 min. The agonist was then removed, and cells were chased in the presence of the drugs for a total of 15 min at 37 ºC. After the incubation period, cells were fixed without permeabilization in 4% PFA for 15 min on ice. Subsequently, surface GluA1-containing receptors were labeled by saturating amount of goat anti-rabbit Alexa-568–conjugated secondary antibody (1:100) for 1 h at 37 °C, followed by permeabilization of cells with 0.1% Triton X-100 for 30 min at room temperature. The endocytosed GluA1-containing receptors were then stained with goat anti-rabbit Alexa-647–conjugated secondary antibody (1:750) for 1 h at 37 °C. Staining of GFP was performed by incubation with the mouse anti-GFP primary antibody (1:500) for overnight at 4 °C, followed by application of the goat anti-mouse Alexa-488–conjugated secondary antibody (1:750) for 1 h at 37 °C. Coverslips were mounted on glass slides and scanned under the confocal microscope. Again, in order to make sure that the Alexa-647–conjugated secondary antibody did not label any detectable surface receptors in our assays, we determined the saturating concentration of the first secondary antibody, that is, Alexa-568–conjugated secondary antibody through a control experiment similar to that we have described in our earlier studies (32, 33).

### Colocalization assay

To determine if different mutants of PICK1 were expressed and targeted properly at the synapse, the extent of colocalization of these mutants with the presynaptic protein Bassoon was measured. Bassoon is a core component of the active zone which acts as a marker to identify synaptic terminals (35). Briefly, different GFP-tagged PICK1 mutants were transfected in primary hippocampal neurons, and colocalization assays were performed on 12 to 14 DIV. Cells were fixed with ice-cold 4% PFA on ice for 15 min. After that, cells were permeabilized using 0.1% Triton X-100 for 30 min at room temperature. Cells were then stained with the anti-PICK1 primary antibody (1:500, in case of endogenous PICK1 staining) or anti-GFP primary antibody (1:500, in case of stating of the GFP-tagged PICK1 constructs) and anti-Bassoon primary antibody (1:500) for overnight at 4 °C. Following primary antibody staining, cells were stained with appropriate secondary antibodies conjugated with Alexa-488 (1:700) to visualize the endogenous PICK1 and PICK1 constructs and Alexa-568 (1:700) to visualize Bassoon for 1 h at 37 °C. Finally, coverslips were mounted on glass slides and imaged under the confocal microscope.

### Western blot analysis and co-immunoprecipitation assay

To check the knockdown efficiency of endogenous PICK1 by shPICK1 as well as various shPICK1-containing replacement constructs of PICK1, Western blot assays were performed. For these assays, primary neurons transfected with the appropriate constructs were washed with ice-cold 1× PBS and lysed in RIPA lysis buffer having protease inhibitor cocktail. Samples were subsequently boiled in 5× Laemmli sample buffer at 99 °C for 10 min and run on SDS-PAGE by loading equal amount of protein in each lane. They were then transferred onto a poly(vinylidene fluoride) membrane and blocked with 5% skimmed milk in 0.05% phosphate-buffered saline with Tween 20 for 1 h at room temperature. The membrane was then incubated with anti-PICK1 antibody (1:500) and anti-β-actin antibody (1:1000) at 4 °C overnight. β-actin was used as the loading control. After washing, membranes were incubated with the HRP-conjugated secondary antibodies (1:5000) for 45 min at room temperature. Blots were developed using femtoLUCENT plus-HRP kit, and images were acquired in ImageQuant LAS 4000.

The ability of mGluR1 and mGluR5 to upregulate the phosphorylation of ERK1/2 upon activation by the agonist was investigated by transfecting the primary neurons with empty vector or shPICK1. Both control cells and shPICK1-transfected cells were preincubated with 100 μg/ml cycloheximide for 5 h to inhibit the synthesis of new receptors. The role of mGluR1 alone in the upregulation of the phosphorylation of ERK1/2 was studied by incubating the cells with the mGluR5 antagonist, MTEP (100 μM), along with 100 μg/ml cycloheximide. Subsequently, 100 μM R,S-DHPG was applied for 5 min in both control cells and shPICK1-transfected cells to initiate the endocytosis of mGluRs. In both the conditions, one set of cells were fixed after 5 min of application of 100 μM R,S-DHPG to measure the extent of upregulation of ERK1/2 phosphorylation by the mGluRs, when they were initially present at the cell surface. The other set of cells were chased for 2.5 h, in absence of the agonist. Subsequently, 100 μM R,S-DHPG was applied again for 5 min, followed by fixation of cells. Both sets of cells were then lysed in RIPA lysis buffer having protease inhibitor cocktail. Samples were subsequently boiled in 5× Laemmli sample buffer at 99 °C for 10 min and run on SDS-PAGE by loading equal amount of protein in each lane. They were then transferred onto a poly(vinylidene fluoride) membrane and blocked with 5% skimmed milk in 0.05% phosphate-buffered saline with Tween 20 for 1 h at room temperature. The phospho-ERK1/2 and total ERK1/2 immunoblottings were performed using anti-phospho-p44/42 MAPK (ERK1/2) antibody (1:1000) and anti-p44/42 MAPK (ERK1/2) antibody (1:1000), respectively. After washing, membranes were incubated with the HRP-conjugated secondary antibodies (1:5000) for 45 min at room temperature. Blots were developed using femtoLUCENT plus-HRP kit, and images were acquired in ImageQuant LAS 4000.

Co-immunoprecipitation experiments were performed to check for the interaction of PICK1 with mGluR1. FLAG-mGluR1 construct was transfected in primary neurons. Seventy two hours post-transfection, cells were treated with 100 μM R,S-DHPG for 5 min. The FLAG-mGluR1–transfected control cells were not treated with the ligand. Subsequently, immunoprecipitation was performed following standard procedures. Briefly, cells were washed with ice-cold PBS and lysed using TAP lysis buffer (20 mM Tris pH 8.0, 150 mM NaCl, 0.5% NP-40, 1 mM MgCl_2_, 1 mM Na_3_VO_4_, 1× protease inhibitor cocktail). The lysate was centrifuged at 15,000 rpm for 30 min. Then, 50 μl of supernatant was collected as input. Immunoprecipitation was performed by incubating the remaining supernatant with protein A/G beads that were prepared by overnight incubation with anti-FLAG primary antibodies. After 6 to 8 h, beads were washed and samples were boiled in 2× Laemmli buffer post elution. The samples were run on SDS-PAGE followed by Western blotting using the method as described above. For immunoblotting, antibodies against FLAG (1:750) and PICK1 (1:500) were used.

### Image acquisition and analysis

Cells were imaged in Zeiss LSM 780 confocal laser scanning microscope using a 63× oil immersion objective (NA = 1.4). Each experiment was repeated at least three times. Images from all the conditions in a particular experiment were obtained using identical parameters. All the analyses procedures have been described in our previous studies (32, 33). Raw images were used for all analyses, and quantitation was done using ImageJ software (https://imagej.nih.gov/ij/download.html) (NIH) (45). Briefly, raw images were maximally projected and thresholded using identical values for a particular experiment. The thresholded areas occupied by the fluorescence of the labeled surface and internalized receptors were subsequently measured. The internalization index was then calculated by dividing the value contributed by the internal fluorescence with the value contributed by the total fluorescence (surface + internal). These values were then normalized with respect to their controls. In order to measure the surface receptors in all our assays, surface fluorescence was divided by the cell area, which was determined by measuring background fluorescence using a low threshold level. These values were then normalized to the average surface fluorescence of control cells. All the data represents dendritic values of primary hippocampal neurons, which were defined by the area 10 μm away from the soma. The quantitation of all the experiments has been represented as a combination of results from all three repeats of that particular experiment. The raw images were processed in Adobe Photoshop software (https://www.adobe.com/in/products/photoshop.html) using identical values of brightness and contrast to obtain the representative images. The western blots and co-immunoprecipitation experiments were also quantified using ImageJ software.

### Statistical analysis

Data are presented as mean ± SD. Experimental group results were compared with each other using Student’s *t* test or one-way ANOVA followed by Tukey’s post-test. A *p* value of > 0.05 was considered as nonsignificant.

## Data availability

All data are contained within the manuscript.

## Supporting information

This article contains supporting information.

## Conflict of interest

The authors declare that they have no conflicts of interest with the contents of this article.
