# Supplementary material for: A vital role for PICK1 in the differential regulation of metabotropic glutamate receptor internalization and synaptic AMPA receptor endocytosis
Source: J Biol Chem. 2023 May 18;299(6):104837. doi: 10.1016/j.jbc.2023.104837 (PMC10276164; doi:10.1016/j.jbc.2023.104837)
Supplement: Supporting information [file mmc1.pdf]

Supporting Information

A vital role for PICK1 in the differential regulation of metabotropic glutamate receptor internalization and synaptic AMPA receptor endocytosis

Namrata Ramsakha, Prachi Ojha, Subhajit Pal, Sanjeev Routh, Ami Citri and Samarjit Bhattacharyya

List of materials included:

1. Figure S1

2. Figure S2

3. Table of p values

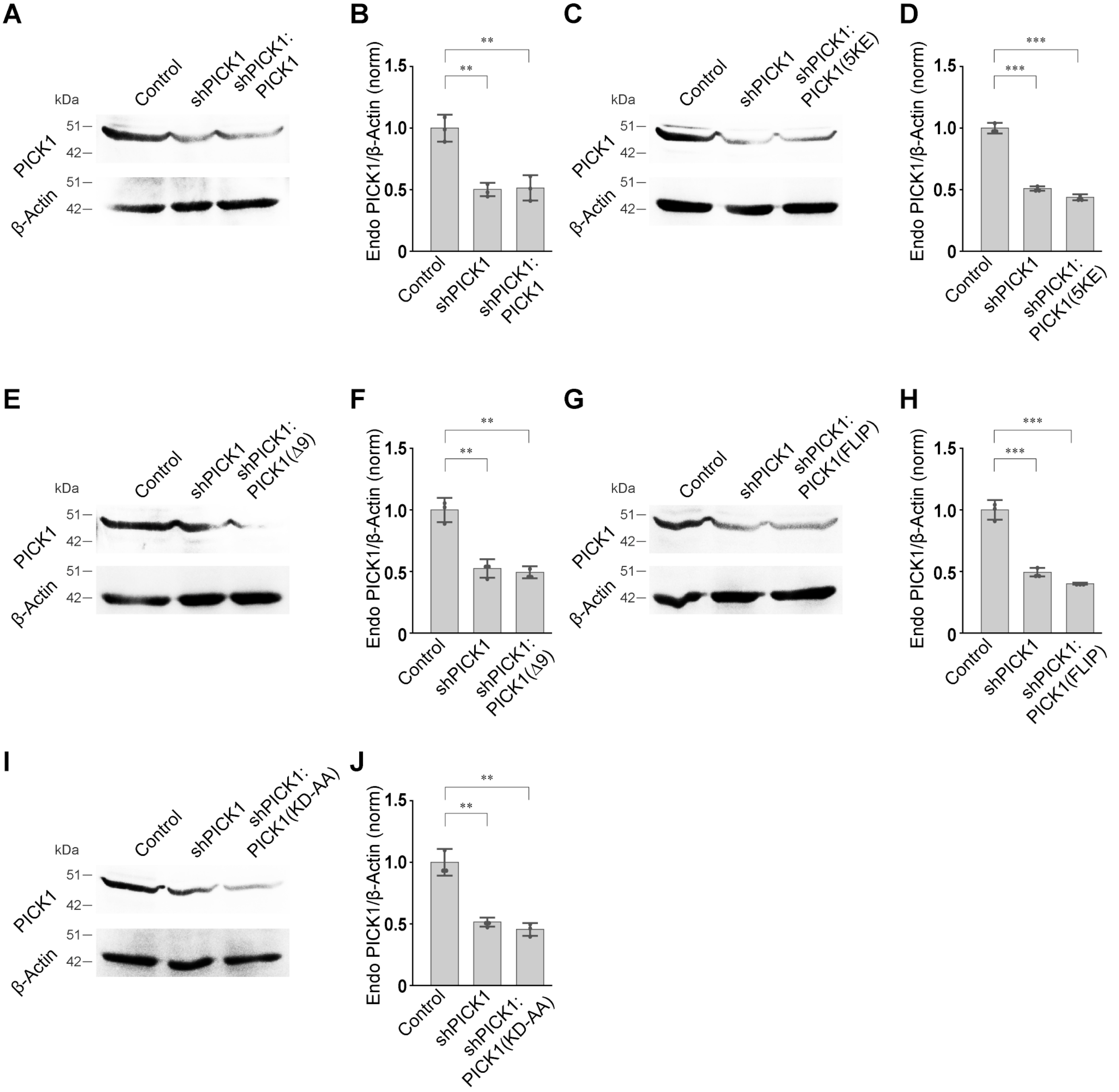
A
Control
shPICK1
shPICK1:
PICK1
kDa
51
42
PICK1
β-Actin
B
Endo PICK1/β-Actin (norm)
1.5
1.0
0.5
0
**
**
Control
shPICK1
shPICK1:
PICK1
C
Control
shPICK1
shPICK1:
PICK1(5KE)
D
***
***
shPICK1:
PICK1(5KE)
E
shPICK1:
PICK1(Δ9)
F
G
shPICK1:
PICK1(FLIP)
H
I
shPICK1:
PICK1(KD-AA)
J

Figure S1

Figure S1. Knockdown of endogenous PICK1 by shPICK1 and PICK1 replacement constructs. (A, B) Western blot (A) and quantitation of western blots (B), showing knockdown of the endogenous PICK1 by shPICK1 and full-length PICK1 replacement construct (N = 3). (C, D) Acute knockdown of endogenous PICK1 by shPICK1 and PICK1(5KE) replacement construct, as shown by the western blot (C) and quantitation of the western blots (D) (N = 3). (E, F) Western blot (E) and quantitation of western blots (F) showing the acute knockdown of endogenous PICK1 by shPICK1 and PICK1(Δ9) replacement construct (N = 3). (G, H) Acute knockdown of endogenous PICK1 by shPICK1 and PICK1(FLIP) replacement construct represented by western blot (G) and quantitation of western blots (H) (N = 3). (I, J) Representative western blot (I) and quantitation of western blots (F) showing the knockdown of endogenous PICK1 by shPICK1 and PICK1(KD-AA) replacement construct (N = 3). Results are presented as means ± SD. ***, $p < 0.001$; **, $p < 0.01$.

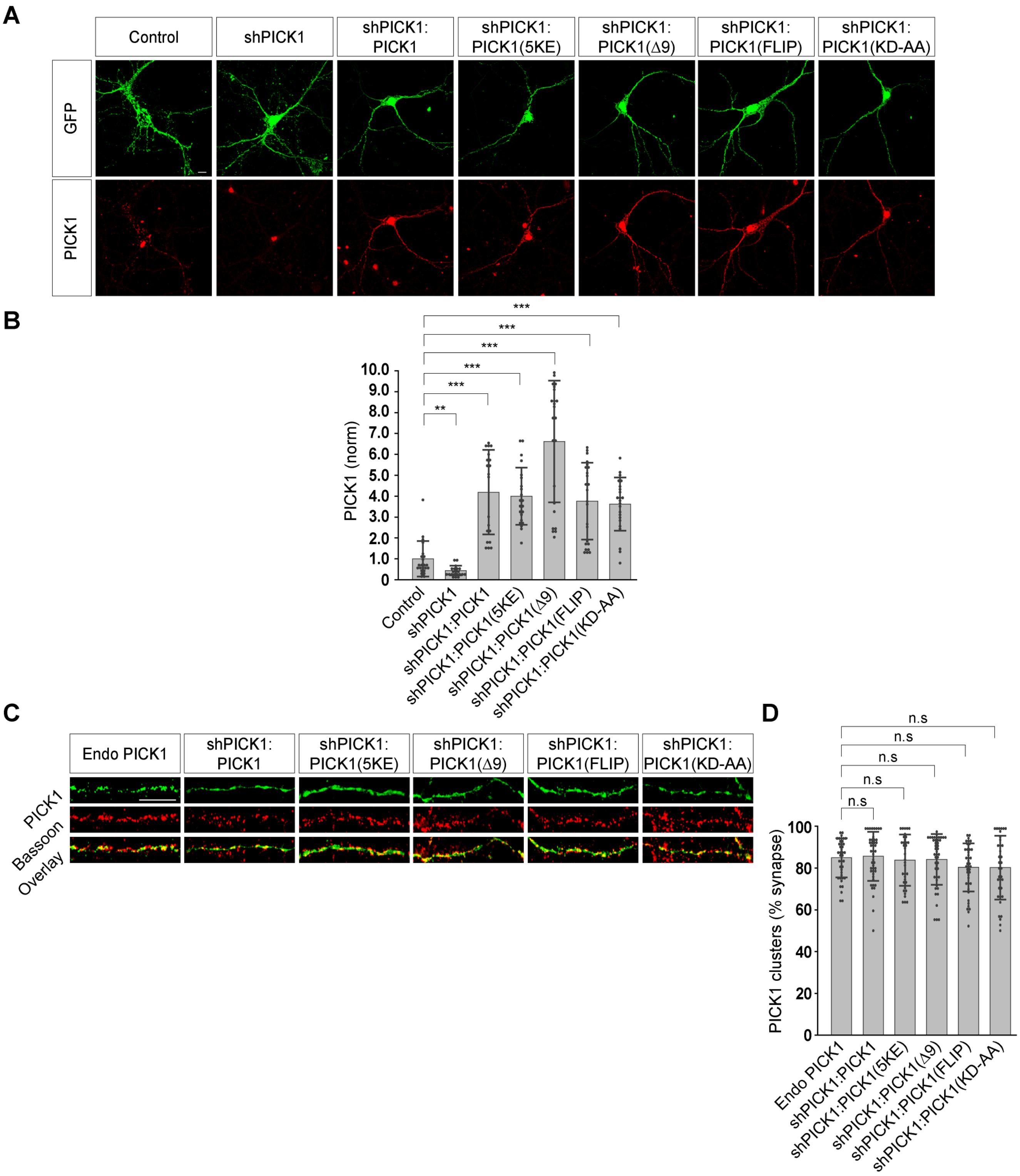
A
Control
shPICK1
shPICK1: PICK1
shPICK1: PICK1(5KE)
shPICK1: PICK1(Δ9)
shPICK1: PICK1(FLIP)
shPICK1: PICK1(KD-AA)
GFP
PICK1
B
PICK1 (norm)
10.0
9.0
8.0
7.0
6.0
5.0
4.0
3.0
2.0
1.0
0
**
***
Control
shPICK1
shPICK1:PICK1
shPICK1:PICK1(5KE)
shPICK1:PICK1(Δ9)
shPICK1:PICK1(FLIP)
shPICK1:PICK1(KD-AA)
C
Endo PICK1
shPICK1: PICK1
shPICK1: PICK1(5KE)
shPICK1: PICK1(Δ9)
shPICK1: PICK1(FLIP)
shPICK1: PICK1(KD-AA)
PICK1
Bassoon
Overlay
D
n.s
PICK1 clusters (% synapse)
100
80
60
40
20
0
Endo PICK1
shPICK1:PICK1
shPICK1:PICK1(5KE)
shPICK1:PICK1(Δ9)
shPICK1:PICK1(FLIP)
shPICK1:PICK1(KD-AA)

Figure S2

Figure S2. Expression and synaptic localization of PICK1 constructs. (A, B) Representative images (A) and quantitation (B) showing that shPICK1 knocked down the endogenous PICK1 and all the replacement constructs used in this study viz., wild-type PICK1, PICK1(5KE), PICK1(Δ9), PICK1(FLIP) and PICK1(KD-AA) expressed properly (Number of dendrites: control = 23; shPICK1 = 21; shPICK1:PICK1 = 20; shPICK1:PICK1(5KE) = 22; shPICK1:PICK1(Δ9) = 23; shPICK1:PICK1(FLIP) = 24; shPICK1:PICK1(KD-AA) = 23) (For immunostaining following antibodies were used: in the upper panels of (A) antibodies against GFP were used for all the conditions and for the lower panels of (A) antibodies against PICK1 were used for all the conditions). (C) Representative images showing the colocalization of Bassoon with various mutants of PICK1 (For immunostaining following antibodies were used: in the upper left panel of endo PICK1 condition antibody against PICK1 was used and in all other conditions in the upper panels antibodies against GFP were used. For the middle panels, antibodies against Bassoon were used for all the conditions). All the constructs of PICK1 were observed to colocalize with Bassoon similar to endogenous PICK1. (D) Quantitation also suggested that all the constructs of PICK1 were localized at the synapse to the similar extent compared to endogenous PICK1 (Number of dendrites: endo PICK1 = 40; shPICK1:PICK1 = 45; shPICK1:PICK1(5KE) = 35; shPICK1:PICK1(Δ9) = 45; shPICK1:PICK1(FLIP) = 41; shPICK1:PICK1(KD-AA) = 35). Results are presented as means ± SD. Scale bar, 10 μm. ***, $p < 0.001$; **, $p < 0.01$; n.s, $p > 0.05$.

p values

Figure	Groups	p value
Figure 1C	Between control and shPICK1	5.926×10^{-15}
	Between control and shPICK1:PICK1	0.705
Figure 1E	Between control and control + DHPG	1.054×10^{-13}
	Between control + DHPG and shPICK1 + DHPG	1.723×10^{-10}
	Between control + DHPG and shPICK1:PICK1 + DHPG	0.739
Figure 1G	Between control and DHPG	0.775
Figure 2C	Between control and shPICK1	5.325×10^{-11}
	Between control and shPICK1:PICK1(5KE)	3.845×10^{-10}
Figure 2E	Between control and control + DHPG	3.832×10^{-17}
	Between control + DHPG and shPICK1 + DHPG	6.887×10^{-16}
	Between control + DHPG and shPICK1:PICK1(5KE) + DHPG	7.69×10^{-15}
Figure 3C	Between control and shPICK1	2.975×10^{-9}
	Between control and shPICK1:PICK1(Δ9)	9.966×10^{-15}
Figure 3E	Between control and control + DHPG	3.142×10^{-24}
	Between control + DHPG and shPICK1 + DHPG	1.882×10^{-18}
	Between control + DHPG and shPICK1:PICK1(Δ9) + DHPG	6.457×10^{-14}
Figure 4C	Between control and shPICK1	2.664×10^{-13}
	Between control and shPICK1:PICK1(FLIP)	1.033×10^{-14}
Figure 4E	Between control and control + DHPG	6.077×10^{-12}
	Between control + DHPG and shPICK1 + DHPG	6.839×10^{-14}
	Between control + DHPG and shPICK1:PICK1(FLIP)	1.023×10^{-10}

	+ DHPG	
Figure 5C	Between control and shPICK1	1.221×10^{-15}
	Between control and shPICK1:PICK1(KD-AA)	1.213×10^{-14}
Figure 5E	Between control and control + DHPG	8.115×10^{-12}
	Between control + DHPG and shPICK1 + DHPG	7.983×10^{-14}
	Between control + DHPG and shPICK1:PICK1(KD-AA) + DHPG	6.809×10^{-8}
Figure 6B	Between untreated and DHPG	0.004
	Between 2.5 hr untreated and 2.5 hr DHPG	0.011
Figure 6D	Between untreated and DHPG	0.001
	Between 2.5 hr untreated and 2.5 hr DHPG	0.008
Figure 6F	Between untreated and DHPG	1.104×10^{-13}
	Between 2.5 hr untreated and 2.5 hr DHPG	4.8×10^{-11}
	Between 2.5 hr (OA + FK-506) untreated and 2.5 hr (OA + FK-506) DHPG	0.388
Figure 6H	Between untreated and DHPG	1.136×10^{-7}
	Between 2.5 hr untreated and 2.5 hr DHPG	6.224×10^{-15}
	Between 2.5 hr (OA + FK-506) untreated and 2.5 hr (OA + FK-506) DHPG	0.858
Figure 7B	Between control and shPICK1	0.007
	Between control and shPICK1:PICK1	0.315
Figure 7D	Between control and control + DHPG	5.378×10^{-24}
	Between control + DHPG and shPICK1 + DHPG	0.571
	Between control + DHPG and shPICK1:PICK1 + DHPG	0.656

Figure 8B	Between untreated and DHPG	0.033
	Between 2.5 hr untreated and 2.5 hr DHPG	0.028
Figure 8D	Between untreated and DHPG	0.006
	Between 2.5 hr untreated and 2.5 hr DHPG	0.99
Figure 8F	Between untreated and DHPG	2.061×10^{-7}
	Between 2.5 hr untreated and 2.5 hr DHPG	4.3×10^{-9}
	Between 2.5 hr (OA + FK-506) untreated and 2.5 hr (OA + FK-506) DHPG	0.007
Figure 8H	Between untreated and DHPG	3.589×10^{-13}
	Between 2.5 hr untreated and 2.5 hr DHPG	0.341
	Between 2.5 hr (OA + FK-506) untreated and 2.5 hr (OA + FK-506) DHPG	0.596
Figure S1B	Between control and shPICK1	0.002
	Between control and shPICK1:PICK1	0.005
Figure S1D	Between control and shPICK1	5.334×10^{-5}
	Between control and shPICK1:PICK1(5KE)	3.747×10^{-5}
Figure S1F	Between control and shPICK1	0.003
	Between control and shPICK1:PICK1(Δ9)	0.001
Figure S1H	Between control and shPICK1	5.322×10^{-4}
	Between control and shPICK1:PICK1(FLIP)	2.042×10^{-4}
Figure S1J	Between control and shPICK1	0.002
	Between control and shPICK1:PICK1(KD-AA)	0.001
Figure S2B	Between control and shPICK1	0.005
	Between control and shPICK1:PICK1	2.252×10^{-8}
	Between control and shPICK1:PICK1(5KE)	2.699×10^{-11}

	Between control and shPICK1:PICK1(Δ9)	2.388×10^{-11}
	Between control and shPICK1:PICK1(FLIP)	4.839×10^{-8}
	Between control and shPICK1:PICK1(KD-AA)	1.962×10^{-10}
Figure S2D	Between endo PICK1 and wt-PICK1	0.768
	Between endo PICK1 and PICK1(5KE)	0.642
	Between endo PICK1 and PICK1(Δ9)	0.75
	Between endo PICK1 and PICK1(FLIP)	0.052
	Between endo PICK1 and PICK1(KD-AA)	0.11
